# Effects of Mixed Fermentation with Indigenous Xinjiang Non-*Saccharomyces* Yeasts and *Saccharomyces cerevisiae* on Co-Fermentation Characteristics and the Quality of Munage Wine

**DOI:** 10.3390/foods15162928

**Published:** 2026-08-20

**Authors:** Ying Deng, Yun Wu, Tiancong Zheng, Guiyin Wei, Zhenling Zeng, Huazhou He, Reziyemu Abuduaili, Jiang Yu, Zhenzhen Zhang, Wenrui Ma

**Affiliations:** College of Food Science and Pharmacy, Xinjiang Agriculture University, Urumqi 830052, China

**Keywords:** non-*Saccharomyces* yeasts, mixed fermentation, sequential inoculation, Co-fermentation characteristics, Munage wine, aromatic quality

## Abstract

This study employed sequential inoculation of native non-brewing yeasts *Kluyveromyces marxianus* YC-T7 and *Pichia kudriavzevii* QC-P3 to co-ferment dry white wine from Munage, a unique fresh-eating grape variety native to Xinjiang, providing technical support for the development of regionally distinctive wines in Xinjiang. To investigate how mixed fermentation with indigenous Xinjiang non-*Saccharomyces* (NS) yeasts and *Saccharomyces cerevisiae* (SC) influences the aromatic profile of Munage wine, two NS strains—*K. marxianus* YC-T7 and *P. kudriavzevii* QC-P3—previously isolated and selected by our group were sequentially co-inoculated with *S. cerevisiae* CEC01. Strain growth dynamics were first characterized in a synthetic grape juice (SGJ) system under varying inoculation protocols; subsequently, Munage grapes were fermented under optimized sequential inoculation to assess physicochemical parameters, volatile aroma composition, and sensory attributes of the resulting dry white wine. Results demonstrated that, under sequential inoculation, the QC-P3 + SC consortium entered the late logarithmic phase on day 4 and achieved a final ethanol concentration of 10.29%vol, whereas the YC-T7 + SC consortium reached the same growth stage on day 5 with 10.27%vol ethanol—confirming robust and timely alcoholic fermentation for both consortia. Consequently, sequential inoculation was adopted as the standard protocol for Munage wine production. All key physicochemical parameters complied with China’s national standards for dry white wine. Relative to pure SC fermentation, the YC-T7 + SC treatment increased tartaric acid, α-ketoglutaric acid, and citric acid contents by 3.15%, 12.16%, and 2.18%, respectively; the QC-P3 + SC treatment induced more pronounced enhancements—22.58%, 51.64%, and 14.70%, respectively. A total of 54 volatile compounds were identified, including 29 esters, 12 alcohols, 7 organic acids, 4 phenolic compounds, and 2 others. Integrated analysis using odor activity values (OAVs), principal component analysis (PCA), and descriptive sensory evaluation consistently revealed that the YC-T7 + SC wine exhibited the highest concentrations of fruit- and flower-associated esters, while the QC-P3 + SC wine was distinguished by a distinctive pineapple-like aroma driven predominantly by ethyl butyrate. Collectively, sequential co-inoculation of indigenous Xinjiang NS strains YC-T7 and QC-P3 with *S. cerevisiae* significantly improved Munage wine quality—particularly in acidity balance, polyphenol retention, and aromatic complexity—thereby offering both novel microbial resources and a validated technical framework for stylistically authentic vinification of terroir-expressive wines from the Xinjiang region.

## 1. Introduction

Chinese wine-producing regions are widely distributed, ranging from Yantai, Shandong Province and Changli, Hebei Province in eastern China to the eastern foot of Helan Mountain in Ningxia, the Hexi Corridor in Gansu, and the northern and southern foothills of the Tianshan Mountains in Xinjiang in northwest China, forming a golden latitude belt for wine grapes with unique terroir characteristics. Among these regions, Xinjiang, benefiting from a long sunshine duration, large diurnal temperature difference and low rainfall, has developed four major producing areas: the Northern Foothills of Tianshan Mountains, the Ili River Valley, the Yanqi Basin and the Turpan-Hami Basin, and has become China’s largest production base for bulk wine. The main wine grape varieties cultivated in Xinjiang include common varieties such as Cabernet Sauvignon, Merlot and Chardonnay, in addition to a unique table grape variety, the most representative of which is Munage grape, with the scientific name *Vitis vinifera* L. cv. Munage. It belongs to the *Vitis vinifera* species of Eurasian origin, and is also called Munage or Gobi grape. Native to Artux, Xinjiang, it was later introduced to multiple oases in the Tarim Basin south of the Tianshan Mountains (such as Kashi and Hotan), with the highest quality from the Artux region. Munage grapes have full, neatly arranged oval berries with thick, tenacious yellow–green peels. The berries feature a high sugar content [1], well-balanced sugar–acid ratio, full berry shape, and good tolerance to storage and transportation. In terms of flavor characteristics, Munage is rich in aroma precursors such as terpenes, with abundant potential aromatic resources [2], and has distinct potential for fruity and floral aroma styles [3]. Currently, Xinjiang Munage grapes are primarily consumed fresh, with insufficient development of downstream deep-processed products. Few studies have been conducted on fermented products such as dry white wine, which restricts the extension of their industrial chain and the improvement of their added value.

**Figure 1:**
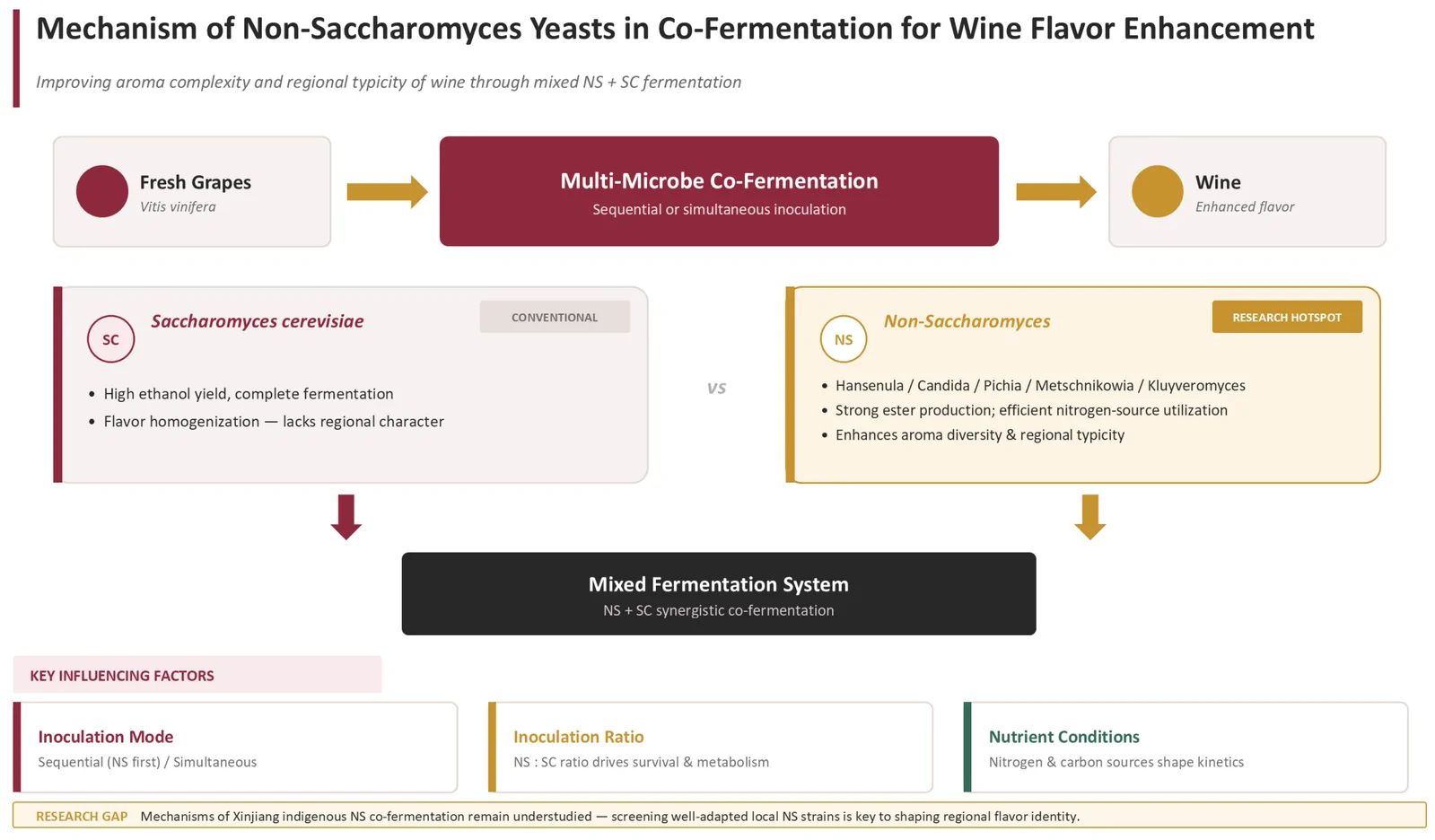
Mechanism of mixed fermentation by non-*Saccharomyces* yeasts.

Wine is an alcoholic beverage produced by the multi-microbe co-fermentation of fresh grapes, and its flavor quality is closely correlated with the metabolic characteristics of fermentation strains [4]. Currently, industrial wine production primarily relies on *Saccharomyces cerevisiae* (SC) as the starter culture, which features a high ethanol conversion efficiency and complete fermentation. However, the relatively concentrated metabolic pathway of a single starter strain leads to a homogenization trend in the flavor characteristics of wines originating from different regions and produced from different grape cultivars [5]. With the increasing consumer demand for diverse wine flavors and regional typicity, the application of Non-*Saccharomyces* yeasts (NS) to improve the flavor quality of wine has emerged as a research hotspot in recent years. At present, common non-*Saccharomyces* yeast genera in winemaking include *Hanseniaspora*, *Candida*, *Pichia*, *Metschnikowia*, *Kluyveromyces* and others [6]. Generally, non-*Saccharomyces* yeasts require co-inoculation with *Saccharomyces cerevisiae* to complete alcoholic fermentation, and can produce a larger amount of aroma compounds, as shown in Figure 1. Linbo Li et al. [7] conducted co-inoculation fermentation of Sauvignon blanc dry white wine with *Torulaspora delbrueckii*, *Hanseniaspora uvarum* and *Saccharomyces cerevisiae*, and found that mixed-culture fermentation significantly increased the total ester content, especially acetate esters, endowing the wine with distinct tropical fruit and citrus aroma notes; Yu Chen et al. [8] carried out mixed-culture fermentation of apricot wine using indigenous *Saccharomyces cerevisiae* and *Pichia kudriavzevii*, and revealed that mixed-culture fermentation effectively increased the contents of fruity esters and terpenes, and improved the flavor and aroma complexity of the resulting wine. *K. marxianus* can significantly increase the content of fruity esters such as isoamyl acetate and ethyl hexanoate during mixed fermentation, thereby enhancing the fruitiness and flavor complexity of wine [9]. By contrast, *P. kudriavzevii* has a superior ester-producing capacity and nitrogen source utilization properties, which can enrich the ester aroma hierarchy of wine and affect fermentation kinetics [10,11]. The co-fermentation characteristics between NS and *S. cerevisiae* are affected by multiple factors including the inoculation method, inoculation ratio [12], and nutrient conditions. The inoculation method exerts a critical influence on the survival time and metabolic activity of NS. Sequential inoculation enables NS to complete the main proliferation before *S. cerevisiae* is inoculated [13], which is conducive to the accumulation of flavor metabolites, while simultaneous inoculation refers to the co-inoculation of *S. cerevisiae* and NS before fermentation to complete fermentation together. Currently, there is scarce research on the growth interaction mechanism of indigenous non-*Saccharomyces* yeasts from Xinjiang in mixed fermentation systems and their effects on wine quality. Therefore, screening and utilizing indigenous non-*Saccharomyces* yeasts with good adaptability to regional grape cultivars is of great value for shaping the flavor quality of wines with regional characteristics.

Currently, classic dry white wines primarily rely on commercial wine yeasts fermented with international grape varieties such as Chardonnay and Riesling. However, in research on producing dry white wines using native non-*Saccharomyces* (NS) yeasts paired with local grape varieties in Xinjiang, two strains of native non-*Saccharomyces cerevisiae* isolated from Xinjiang, namely *K. marxianus* YC-T7 and *P. kudriavzevii* QC-P3, which were pre-screened by our research group, were selected as research objects. A simulated grape juice system was adopted to systematically investigate the co-fermentation characteristics between non-*Saccharomyces cerevisiae* (NS) and *S. cerevisiae* CEC01 under co-inoculation and sequential inoculation strategies, as well as the influence of *Saccharomyces cerevisiae* metabolites on the co-fermentation characteristics’ effect. On this basis, using Munage grapes as raw materials, mixed-culture fermentation of Munage dry white wine was carried out with YC-T7 and QC-P3 respectively, so as to provide a theoretical basis for the development and utilization of native yeast resources in Xinjiang and the quality improvement of Munage wine.

## 2. Materials and Methods

### 2.1. Strains

The non-*Saccharomyces cerevisiae* strains used in this study are two non-*Saccharomyces cerevisiae* strains isolated and screened by our research group in previous work, namely *K. marxianus* YC-T7 and *P. kudriavzevii* QC-P3. The commercial *S. cerevisiae* CEC01 was purchased from the official Taobao flagship store of Angel Yeast Co., Ltd. (Yichang, Hubei Province, China).

### 2.2. Methods

#### 2.2.1. Strain Activation

After thawing glycerol stock-preserved strains, an appropriate volume of bacterial suspension was aspirated and transferred into liquid Yeast Extract Peptone Dextrose Medium (YPD), followed by shake cultivation at 28 °C with a shaking speed of 150 r/min until the strain reached the logarithmic growth phase. After two consecutive passages, solid YPD culture was performed to obtain single colonies.

Activation of primary seed liquid: A single colony was picked and inoculated into YPD medium, followed by shake cultivation at 28 °C until the culture reached the logarithmic growth phase, with an OD_600_ nm of 0.8~1.0.

Activation of secondary seed liquid: 1 mL of the primary seed liquid was aspirated and added to 50 mL of liquid YPD medium, followed by shake cultivation at 28 °C. The activation was terminated when the OD_600_ nm reached 0.8~1.0 and the yeast cell count reached 10^6^ CFU/mL.

Preparation of simulated grape juice system: For 1 L of simulated grape juice, 150 g glucose, 100 g fructose, 2 g yeast extract, 2 g ammonium sulfate, 0.3 g citric acid, 4 g L-malic acid, 5 g L-tartaric acid, 5 g potassium dihydrogen phosphate, 0.4 g magnesium sulfate, 0.2 g sodium chloride and 0.05 g manganese sulfate were mixed thoroughly. After mixing, the pH was adjusted to 3.5 with sodium hydroxide, and the mixture was sterilized for later use. The sterilized simulated grape juice was aliquoted into sterile fermentation bottles as 150 mL systems per bottle.

#### 2.2.2. Kinetic Analysis of Co-Fermentation by Different Strains

Pure culture fermentation: Secondary seed cultures of *S. cerevisiae* CEC01, *K. marxianus* YC-T7 and *P. kudriavzevii* QC-P3 were individually inoculated into simulated grape must for fermentation, with a final yeast cell concentration of 10^6^ CFU/mL, followed by constant temperature incubation at 28 °C. Fermentation was conducted for 7 days, and samples were collected at a fixed time daily to determine the biomass, reducing sugar content and alcohol content.

Mixed culture fermentation: Two inoculation strategies were adopted, namely simultaneous inoculation and sequential inoculation. Simultaneous inoculation: Activated *S. cerevisiae* CEC01 was co-inoculated simultaneously with YC-T7 and QC-P3 respectively into simulated grape must, and fermentation was carried out for 7 days. Sequential inoculation: Activated YC-T7 and QC-P3 were firstly inoculated into simulated grape must and fermented for 48 h, followed by inoculation of *S. cerevisiae* CEC01 and co-fermentation for another 7 days. The inoculation ratio of strains was 1:1, with a total inoculum concentration of 10^6^ CFU/mL. Samples were collected at a fixed time daily for the detection of biomass, reducing sugar content and alcohol content.

Supplementary experiment of adding *Saccharomyces cerevisiae* metabolites: Activated *S. cerevisiae* CEC01 was inoculated into simulated grape must for fermentation. After 3 days of fermentation, the fermentation broth was centrifuged at 10,000 r/min for 10 min, and the supernatant was filtered through a 0.22 μm sterile membrane. The reducing sugar content of the obtained non-*Saccharomyces* fermentation supernatant was measured, and the sugar content was adjusted to the initial concentration of simulated grape must. Secondary seed cultures of YC-T7 and QC-P3 (with a cell concentration of 1 × 10^6^ CFU/mL) were individually inoculated into the *S. cerevisiae* metabolite solution for single-strain fermentation, which lasted for 7 days. The control group was single-strain fermentation of yeast without the addition of *S. cerevisiae* metabolites, and three biological replicates were set for each group. The yeast inoculum concentration was 10^6^ CFU/mL, and all cultures were incubated at a constant temperature of 28 °C. In all experiments, biomass was determined by hemocytometer counting, and the reducing sugar content and alcohol content were detected using a FOSS automatic wine composition analyzer (FOSS Analytical (Hillerød, Denmark)).

#### 2.2.3. Vinification of Munage Wine

Fresh Muzha grapes were selected as raw material, and the processes of destemming, crushing, and gentle pressing were carried out sequentially to obtain grape must. Chemical analysis revealed an initial total sugar content of 156 g/L in the grape juice. White granulated sugar was added at a dosage of 34 g/L to adjust the sugar concentration to 190 g/L, with an expected final alcohol content of 10.5%vol after fermentation. Subsequently, 30 mg/L pectinase was added to the juice and left to stand at 8 °C for 4 h to fully decompose pectin substances, thereby improving juice yield and clarity. After enzymatic treatment, 40 mg/L potassium metabisulfite was added, followed by cold maceration at 8°C for 20 h.

Sequential inoculation was performed: a single strain of wine yeast served as the control group (designated SC), inoculated at 1 × 10^6^ CFU/mL. For the mixed fermentation treatments, two groups were designated YC-T7+SC and QC-P3+SC. First, NS was inoculated at 1 × 10^8^ CFU/mL, followed by the addition of wine yeast 48 h later at 1 × 10^6^ CFU/mL, resulting in a ratio of 100:1 between the two strains. A total of three experimental groups were established, each with three independent replicates.

The wine was fermented at 18°C under static conditions. Weight measurements were taken and recorded every 24 h, and samples were collected on days 1, 3, 5, and 7 based on weight loss. The alcohol content, titratable acidity, total acidity, volatile acidity, pH, and total polyphenols were determined according to general analytical methods for wine and fruit wines GB/T 15038—2006 [14], which is used in the wine industry of the People’s Republic of China.

#### 2.2.4. Determination of Volatile Aroma Compounds

Flavor compounds were analyzed via headspace solid-phase microextraction coupled with gas chromatography–mass spectrometry (HS-SPME-GC-MS). The procedure was performed with minor modifications based on the method reported by Noguerol-Pato et al. [15]. Briefly, 5 mL of wine sample was accurately measured and transferred into a 30 mL headspace vial, followed by the addition of 3.0 g NaCl. The vial was then sealed with a PTFE/silicone septum, and headspace extraction was conducted under constant temperature after the internal standard was added. Upon completion of extraction, the extraction fiber was inserted into the injection port of the gas chromatography for thermal desorption.

GC conditions: DB-WAX capillary column (Santa Clara, CA, USA.) (30 m × 0.25 mm × 0.25 μm); high-purity helium (He) with a purity ≥99.99% was used as the carrier gas; the flow rate was 1 mL/min; splitless injection was adopted within the first 0.5 min, and the split ratio was set to 10∶1 after 1 min; for programmed temperature elevation, the initial temperature of 45 °C was maintained for 2 min, then increased to 230 °C at a rate of 4 °C/min and held for 7 min; and the inlet temperature was 230 °C.

MS conditions: electron ionization (EI) ion source; ion source temperature: 230 °C; ion source voltage: 450 V; transmission line temperature: 230 °C; electron energy: 70 eV; mass scan range: 29–300 amu.

Qualitative and quantitative analysis: The volatile flavor compounds were identified via retrieval against the mass spectrum database of the National Institute of Standards and Technology (NIST) 70, combined with the retention index and retention time of reference substances. Compounds with a matching degree greater than 80% were selected for the qualitative analysis of volatile flavor substances, and quantitative analysis was performed using the internal standard method.

The odor activity value (OAV) was calculated as OAV = C/OT, where C is the mass concentration of the compound (μg/L) and OT is the odor threshold of the compound. The odor thresholds of the volatile compounds were primarily obtained from Noguerol-Pato et al., [15], with supplementary values from Ferreira, V. et al. [16] and Francis, I.L [17]. These thresholds were determined in wine or water/ethanol matrices and are widely accepted in wine aroma research. Compounds with OAV ≥ 1 are considered to have direct contributions to overall aroma, while those with 0.1 ≤ OAV < 1 may contribute synergistically to the aroma profile.

#### 2.2.5. Determination of Organic Acid Content

The contents of organic acids (tartaric acid, malic acid, lactic acid, citric acid, α-ketoglutaric acid, pyruvic acid, acetic acid) in wine samples were determined by ion chromatography (IC). 

Sample Preparation: Wine samples were diluted with an appropriate multiple of ultrapure water (resistivity: 18.2 MΩ·cm) (diluted 10–50 times according to the content of each organic acid to ensure the response value falls within the linear range of the standard curve), filtered through a 0.45 μm aqueous microporous membrane (Millipore, Darmstadt, Germany), and then injected directly.

Chromatographic conditions: An ICS-3000 ion chromatograph (Dionex Corporation, Sunnyvale, CA, USA) equipped with an EG40 automatic eluent generator, a conductivity detector and a Chromeleon 6.80 chromatography workstation was employed for the analysis. The separation was performed on an IonPac AS11-HC anion exchange column (250 mm × 4 mm) equipped with an IonPac AG11-HC guard column (50 mm × 4 mm) and an ASRS-ULTRA anion suppressor. The mobile phase was KOH solution generated by the EG40 automatic generator with gradient elution. The column temperature was 30 °C; the flow rate was 1.0 mL/min; the suppression current was 124 mA; and the injection volume was 25 μL. The gradient elution program of KOH was as follows: the concentration was maintained at 1.1 mmol/L for 0–16 min, increased to 16.5 mmol/L at 29 min, increased to 20 mmol/L at 35 min, increased to 35 mmol/L at 39 min and maintained until 45 min, increased to 50 mmol/L at 50 min, and returned to 1.1 mmol/L at 50.1 min for column equilibration until 55 min.

Standard curve and quantification: Stock solutions of 1000 mg/L were prepared using pure standards of tartaric acid, malic acid, lactic acid, citric acid, α-ketoglutaric acid, pyruvic acid, and acetic acid (purity ≥99%). These were then stepwise diluted with ultrapure water to create a mixed standard working solution with five concentration gradients. Standard curves were constructed by plotting the mass concentrations (mg/L) of each organic acid on the x-axis and peak areas on the y-axis. The linear correlation coefficients (R^2^) for all compounds exceeded 0.999. Qualitative identification of each organic acid was based on retention times compared to those of the reference standards, while quantification was performed using the external standard method combined with standard curve analysis.

#### 2.2.6. Sensory Evaluation Guidelines

The sensory evaluation of this study was conducted in accordance with ISO 13299:2016 [18], Sensory Analysis Methodology—General Guidance for Establishing a Sensory Profile. The evaluation panel consisted of 16 trained assessors (majoring in Viticulture and Enology Engineering at Xinjiang Agricultural University, 8 males and 8 females, aged 20 to 40 years). All panel members had basic wine evaluation competence and completed no less than 20 class hours of sensory training, including training in aroma recognition, mouthfeel identification and descriptive capability.

Sensory evaluation was conducted in individual booths in a dedicated sensory laboratory compliant with the ISO 8589:2007 [19] standard. Each group of wine samples (SC, YC-T7+SC, QC-P3+SC) with a volume of 30 mL was served in standardized ISO wine-tasting glasses, labeled with random three-digit codes. The serving order was balanced following a randomized Latin square design to eliminate order effects. Between consecutive evaluation rounds, panelists rinsed their palates with purified water and neutralized residual aftertaste with unsalted crackers.

The evaluation dimensions include appearance (color and clarity), aroma (intensity, typicality, and layer structure), taste (acidity, balance, aftertaste, and overall harmony), and typical style, with a 0–10 linear scoring scale adopted (0 = extremely poor, 10 = extremely excellent). Each panelist conducted one independent evaluation. One-way analysis of variance (Duncan’s multiple range test, *p* < 0.05) was performed on the sensory data using SPSS 26.0, and radar charts were plotted to intuitively present the sensory characteristics of samples in each group.

### 2.3. Data Processing and Analysis

All samples were analyzed in triplicate, and the results are presented as mean ± standard deviation (mean ± SD). Data were statistically analyzed using IBM SPSS Statistics 26.0 software. First, the Shapiro–Wilk test for normality and Levene’s test for homogeneity of variances were performed on each group. When assumptions for parametric tests were met, one-way analysis of variance (ANOVA) was used to compare differences among groups, followed by Duncan’s multiple range test for post hoc multiple comparisons, with significance set at *p* < 0.05. Different letters indicate significant differences between groups (*p* < 0.05). The odor activity value (OAV) of volatile aroma compounds was calculated as OAV = C/OT, where C is the compound concentration and OT is the odor threshold reported in the literature. Principal component analysis (PCA) was conducted using Origin 2025 software; heatmaps of aroma compound contents were generated via the Hiplot online platform (https://hiplot.com.cn); three-line tables and radar charts were created using Excel 2021.

## 3. Results

### 3.1. Effect of Inoculation Strategy on the Cell Kinetics of Non-Saccharomyces Yeasts

In this study, the growth and co-fermentation kinetic characteristics of *Saccharomyces cerevisiae* CEC01, *K. marxianus* YC-T7, and *P. kudriavzevii* QC-P3 were investigated respectively in a simulated grape juice (SGJ, composition shown in Section 2.2.1) system at a constant temperature of 28 °C, under three fermentation strategies: single fermentation, simultaneous inoculation (inoculum concentration 1 × 10^6^ CFU/mL, inoculation ratio 1:1) and sequential inoculation (non-*Saccharomyces* yeast was firstly inoculated, and *S. cerevisiae* was added 48 h later, with the inoculum concentration of 1 × 10^6^ CFU/mL for each strain).

As shown in Table 1, in the simultaneous inoculation regime, the maximum biomass of the YC-T7+SC group reached 2.82 × 10^7^ CFU/mL on day 4, representing a 38.3% decrease compared with the YC-T7 group. On day 4, the maximum biomass of the QC-P3+SC treatment group reached 2.34 × 10^7^ CFU/mL, which was 11.0% lower than that of the QC-P3 group. This may be attributed to a certain competitive inhibition between *S. cerevisiae* and non-*Saccharomyces* (NS) strains in the simultaneous inoculation system, which is consistent with the general rule that nutrient and niche competition serves as the primary interaction mode in simultaneous inoculation systems [20].

Under the sequential inoculation regime, the maximum biomass of the YC-T7+SC group reached 3.89 × 10^7^ CFU/mL on day 5, at which point the cells were at the late logarithmic phase, showing a 1-day delay compared with simultaneous inoculation. On day 5, the maximum biomass of the QC-P3+SC group reached 2.19 × 10^7^ CFU/mL, with the cells at the late logarithmic phase, which was consistent with the timeline of simultaneous inoculation. The results indicate that mixed-culture fermentation can complete alcoholic fermentation in both inoculation regimes; however, compared with simultaneous inoculation, sequential inoculation can increase the cell biomass of the mixed-culture fermentation system and delay the occurrence of the late logarithmic phase of cell growth.

**Figure 2:**
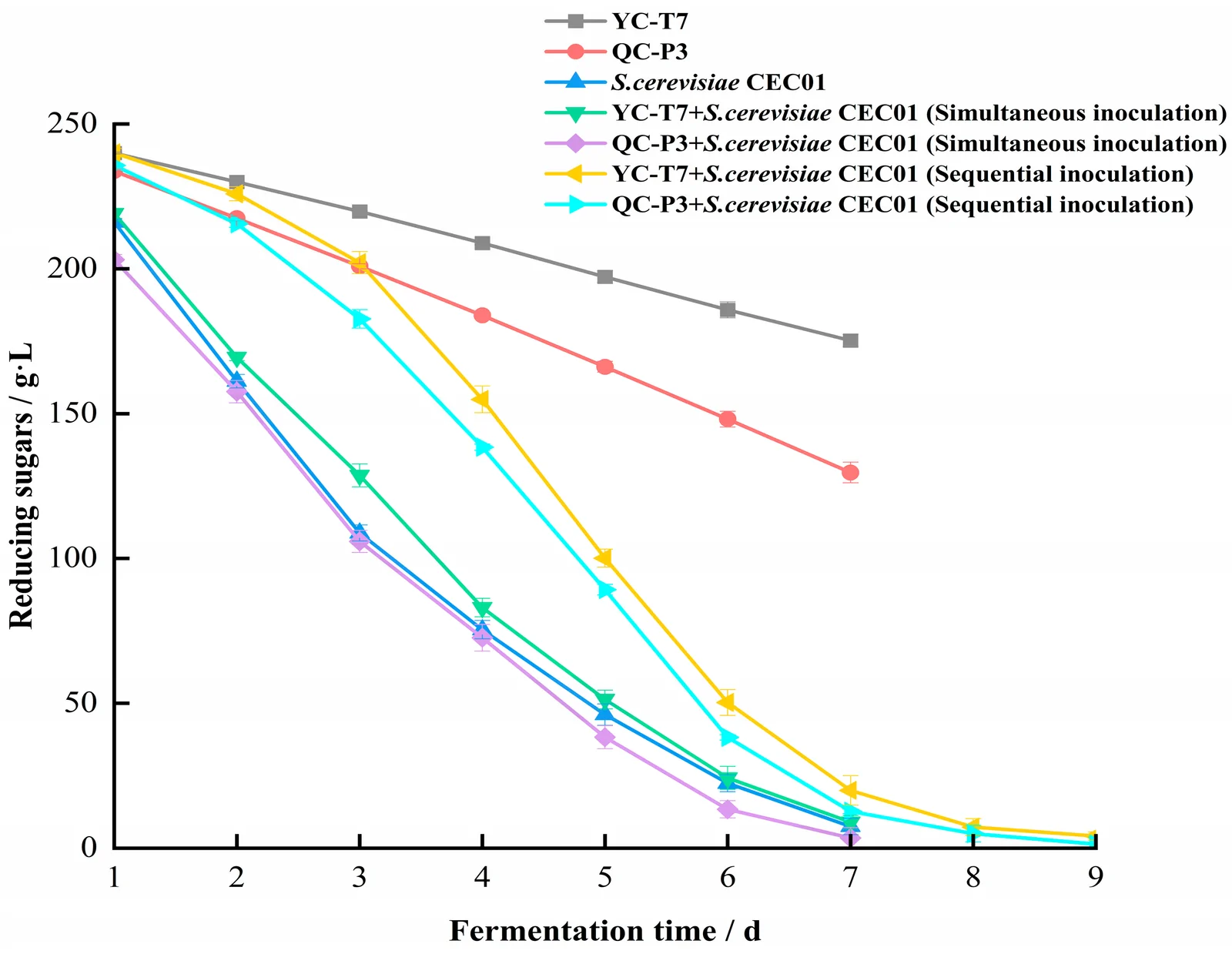
Changes in reducing sugar content during fermentation in synthetic grape juice (SGJ) at 28 °C under different inoculation strategies.

The changes in residual sugar content during mixed-culture fermentation are shown in Figure 2. All samples exhibited a gradual decreasing trend. In single-culture fermentation, the sugar-reducing capacities of YC-T7 and QC-P3 were significantly lower than that of *S. cerevisiae*. At the end of fermentation on day 7, the residual sugar content was 175.25 g/L in the YC-T7 group and 129.69 g/L in the QC-P3 group, while it decreased to 7.50 g/L in the SC group. Previous studies have shown that non-*Saccharomyces cerevisiae* strains generally lack efficient sugar transport systems and alcoholic fermentation pathways, leading to a significantly lower glucose metabolism rate compared to *S. cerevisiae*. After simultaneous inoculation, during the first 3 days of fermentation, the average sugar consumption of the YC-T7+SC group was 45.36 g/L, which was essentially consistent with 46.9 g/L in the single-culture fermentation of SC. However, the residual sugar content decreased slowly on day 4 of fermentation, resulting in a final residual sugar content of 9.01 g/L, which was higher than 7.50 g/L obtained from the single-culture fermentation of SC. At the end of fermentation, the residual sugar consumption of the YC-T7+SC group under simultaneous inoculation was significantly higher than that of the single-culture fermentation of YC-T7.

After sequential inoculation, during the first 5 days of fermentation, the average sugar consumption of the YC-T7+SC group was 37.95 g/L. The residual sugar content decreased slowly on day 7 of fermentation, resulting in a final residual sugar content of 4.2 g/L, which was lower than 9.01 g/L of the YC-T7+SC group obtained via simultaneous inoculation. During the first 5 days of fermentation, the average sugar consumption of the QC-P3+SC group was 36.61 g/L. The residual sugar content decreased slowly on day 7 of fermentation, and the final residual sugar content was 1.42 g/L, which was lower than 3.53 g/L of the QC-P3+SC group obtained via simultaneous inoculation. At the end of fermentation, the residual sugar consumption of both the YC-T7+SC group and the QC-P3+SC group under sequential inoculation was higher than that under simultaneous inoculation and the single-culture fermentation of YC-T7 and QC-P3. On day 5, the maximum biomass of the QC-P3+SC group reached 2.19 × 10^7^ CFU/mL, which corresponded to the late logarithmic phase of cell growth, consistent with the result of simultaneous inoculation. These results indicate that all mixed-culture fermentation systems can complete alcoholic fermentation; however, compared with simultaneous inoculation, sequential inoculation can increase the cell biomass in the mixed-culture fermentation system and delay the entry into the late logarithmic phase of cell growth.

**Figure 3:**
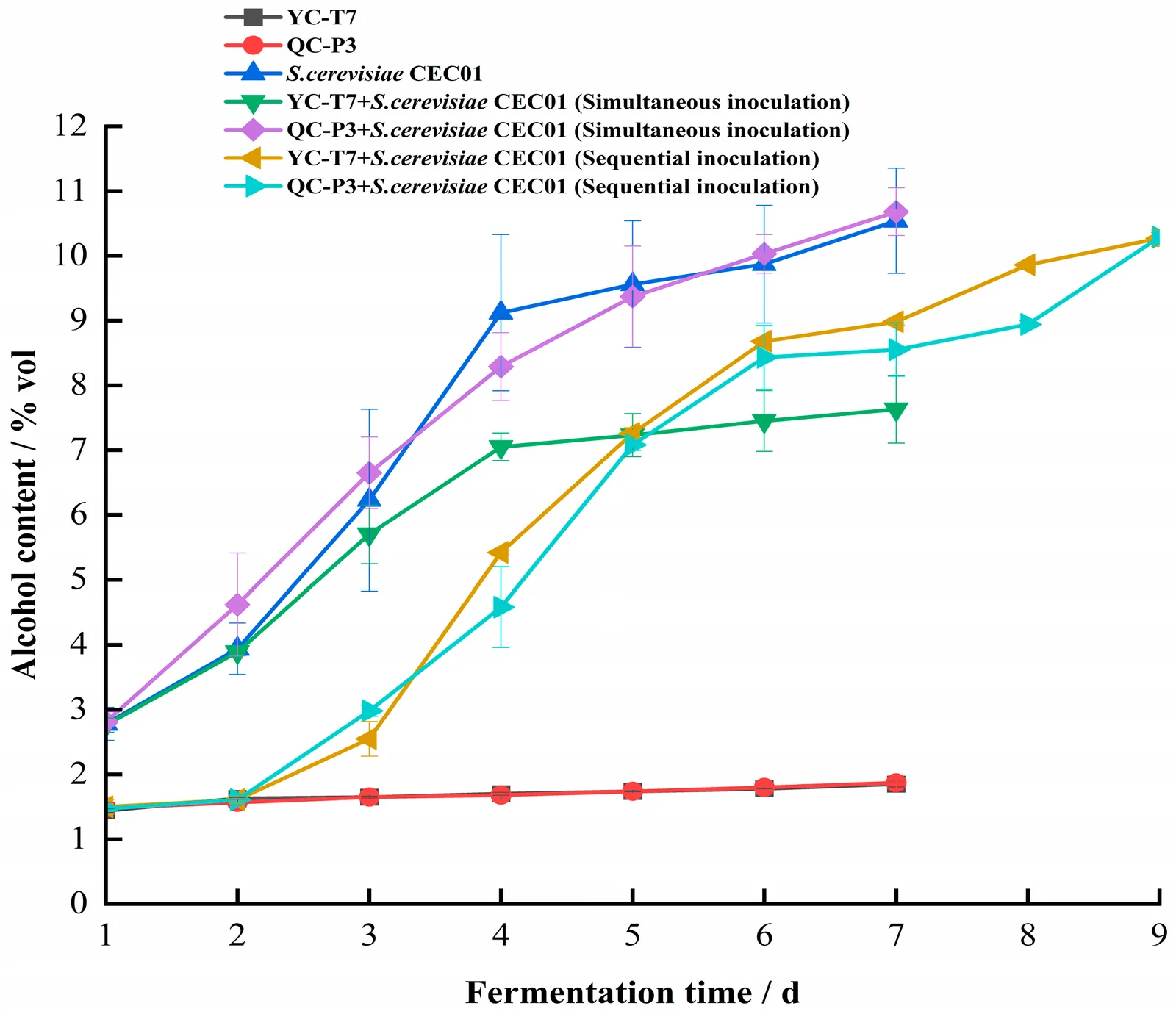
Changes in ethanol content during fermentation in SGJ at 28 °C under different inoculation strategies.

As shown in Figure 3, the alcohol content of all samples exhibits a gradual upward trend. In single-strain fermentation, the alcohol contents of the two non-*Saccharomyces cerevisiae* strains were only 1.85%vol and 1.87%vol at day 7, respectively. Under simultaneous inoculation, the alcohol content of the QC-P3+SC group reached 10.68%vol at day 7, which was 0.14%vol higher than that of single-strain fermentation of SC; the alcohol content of the YC-T7+SC group was 7.63%vol, which was 2.91%vol lower than that of single-strain fermentation of SC, indicating that alcohol production may be inhibited during the co-culture of YC-T7 and SC. Under sequential inoculation, the alcohol content of the QC-P3+SC group was 10.29%vol at day 9, which was 0.25%vol lower than that of the SC group, and the alcohol content of the YC-T7+SC group was 10.27%vol, which was 0.27%vol lower than that of the SC group. During the first 5 days, the average sugar consumption of the QC-P3+SC group was 36.61 g/L. After 7 days of fermentation, the residual sugar decreased slowly, and the final residual sugar was 1.42 g/L, which was lower than 3.53 g/L of the QC-P3+SC group under simultaneous inoculation. At the end of fermentation, the total sugar consumption of both the YC-T7+SC group and the QC-P3+SC group under sequential inoculation was higher than that under simultaneous inoculation and single-strain fermentation of YC-T7 and QC-P3, respectively. At day 5, the maximum biomass of the QC-P3+SC group reached 2.19 × 10^7^ CFU/mL, which corresponds to the late logarithmic phase of cell growth, consistent with the result under simultaneous inoculation. The results demonstrate that all mixed-strain fermentation systems can complete alcoholic fermentation; however, compared with simultaneous inoculation, sequential inoculation can increase the cell biomass of the mixed-strain fermentation system and delay the entry into the late logarithmic phase of cell growth.

### 3.2. Effect of S. cerevisiae Metabolites on the Cell Kinetics of Non-Saccharomyces Yeasts

**Figure 4:**
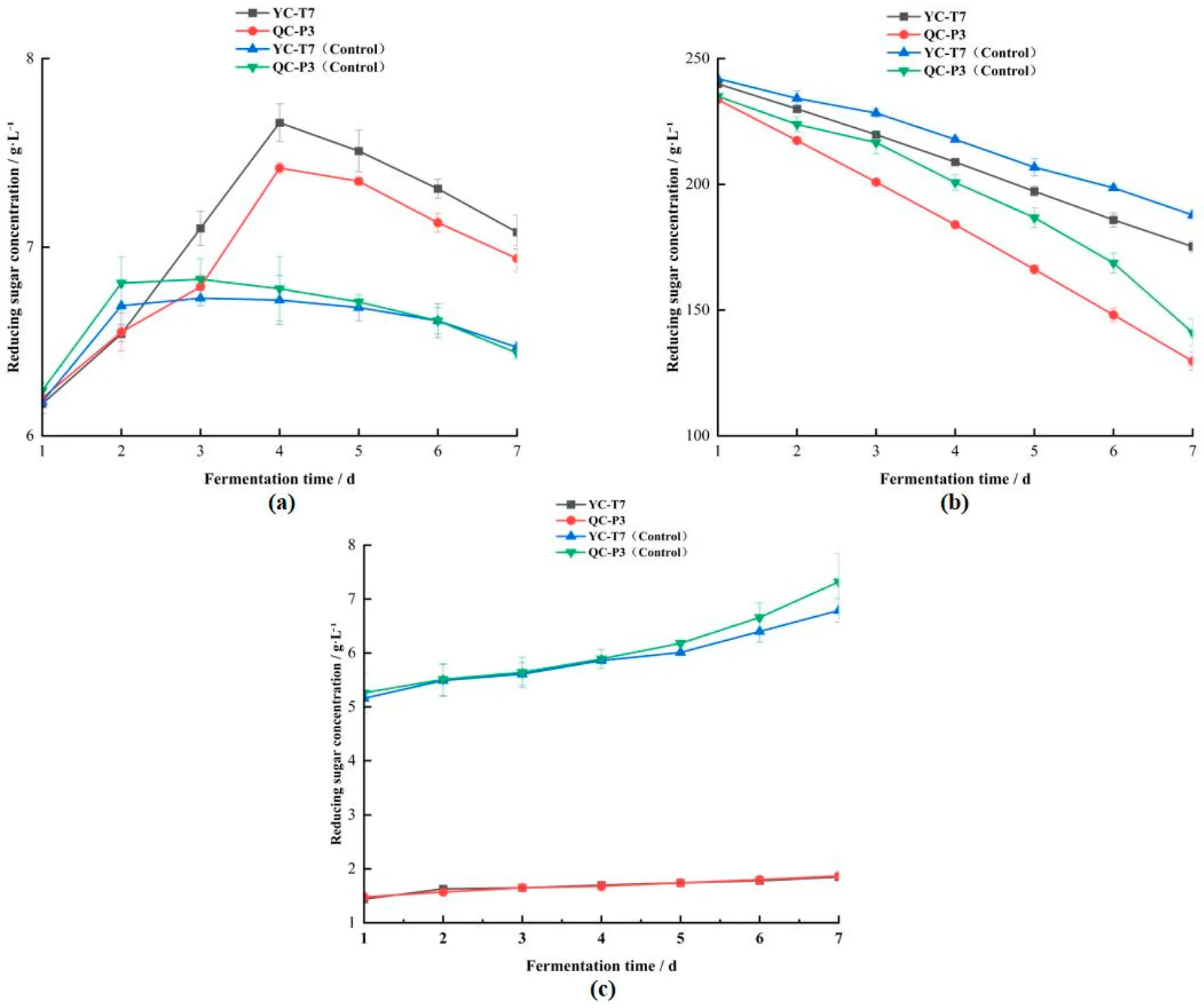
Growth kinetics of non-*Saccharomyces* yeasts in response to *S. cerevisiae* metabolites: (**a**) effect on biomass; (**b**) reducing sugars; and (**c**) ethanol content.

As shown in Figure 4a, after the addition of SC metabolites, the two strains of non-*Saccharomyces cerevisiae* still maintained normal cell growth and stable biomass levels. The overall biomass showed a trend of increasing first and then decreasing. On day 3 of culture, the biomass of the YC-T7 and QC-P3 treatment groups reached their maximum values, which were 5.37 × 10^6^ CFU/mL and 6.76 × 10^6^ CFU/mL respectively. Throughout the entire fermentation period, the viable cell count of the two strains remained at the order of magnitude of 10^6^ CFU/mL, indicating that YC-T7 and QC-P3 still retain cell proliferation capacity in the environment containing SC metabolites.

The changes in residual sugar content are presented in Figure 4b, which shows an overall downward trend. On day 7, the residual sugar contents of the YC-T7 and QC-P3 treatment groups were 187.83 g/L and 141.03 g/L, respectively. This indicates that YC-T7 and QC-P3 continuously consumed reducing sugar throughout the fermentation cycle, and the glucose metabolism activity was not interrupted by the addition of SC metabolites, which can provide a continuous substrate supply for subsequent alcohol production.

The changes in alcohol content are shown in Figure 4c, which exhibits an overall upward trend, and the alcohol content of the treatment groups was significantly higher than that of the control group. On day 1, the alcohol contents of the YC-T7 and QC-P3 treatment groups were 5.16%vol and 5.26%vol, respectively, and increased continuously to 6.79%vol and 7.32%vol on day 7. However, the alcohol contents of the control groups corresponding to YC-T7 and QC-P3 were only 1.85%vol and 1.87%vol respectively on day 7. This demonstrates that in the system containing SC metabolites, YC-T7 and QC-P3 can achieve ethanol production and perform well in alcoholic fermentation.

In conclusion, in the simulated coexistence system containing SC metabolites, YC-T7 and QC-P3 can still maintain stable cell proliferation, a continuous glucose consumption capacity and a sound alcoholic fermentation capacity.

It has been preliminarily verified that the mixed fermentation of YC-T7, QC-P3 and SC can initiate fermentation successfully and complete alcoholic fermentation smoothly, and sequential inoculation outperforms simultaneous inoculation in terms of biomass growth, sugar consumption capacity and alcohol production. Therefore, sequential inoculation will be adopted for the subsequent mixed fermentation of Munage grape wine.

### 3.3. Effect of Mixed Fermentation on the Quality of Munage Wine

#### 3.3.1. CO_2_ Weight-Loss Analysis

**Figure 5:**
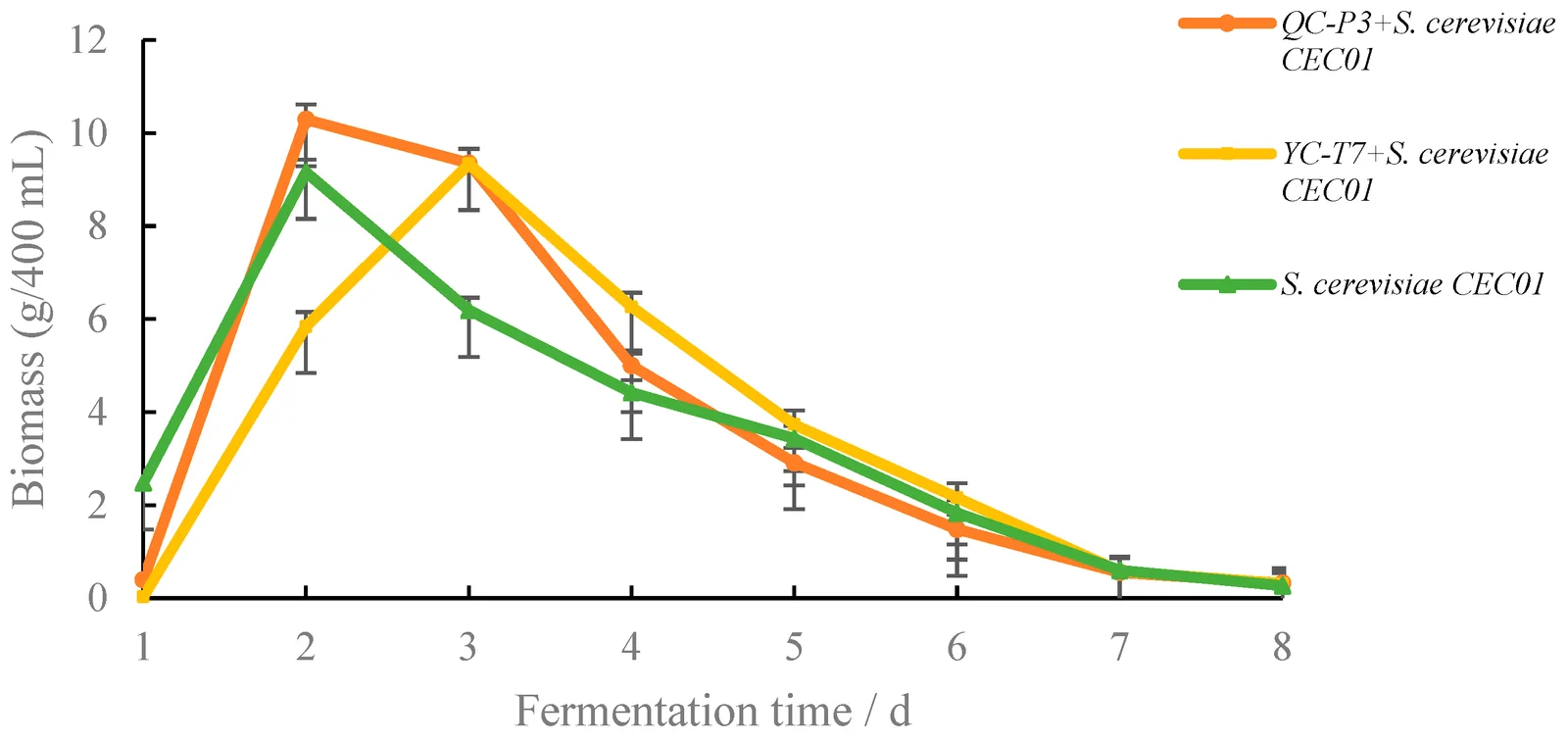
CO_2_ weight-loss profile during Munage wine fermentation at 18 °C.

As shown in Figure 5, the CO_2_ weight loss exhibits a trend of initial increase followed by subsequent decrease. On day 1 of alcoholic fermentation, the weight loss of all groups is insignificant, which corresponds to the fermentation initiation phase. From day 2, the fermentation enters the main fermentation phase: the SC group and the QC-P3+SC group show significant weight loss, reaching 9.16 g and 10.29 g respectively, while the YC-T7+SC group exhibits relatively lagged fermentation progress and reaches its maximum daily weight loss of 9.34 g on day 3. On day 5, the weight loss of all groups tends to stabilize and the fermentation enters the late phase. By day 8, the daily weight loss of the SC group, QC-P3+SC group and YC-T7+SC group decreases to 0.27 g, 0.32 g and 0.31 g respectively, all below 0.5 g, indicating that the fermentation is largely completed.

#### 3.3.2. Basic Physicochemical Indicators of Munage Wine

Upon completion of fermentation, the physicochemical indicators of all samples are presented in Table 2. The ethanol content of the SC group is 10.62%vol, that of the YC-T7+SC group is 10.68%vol, and that of the QC-P3+SC group is 10.74%vol. The residual sugar content of all samples is lower than 4.0 g/L, which meets the standard for dry wines, indicating that the mixed fermentation of YC-T7, QC-P3 and SC can complete alcoholic fermentation, which is consistent with the results of fermentation kinetics and alcohol content presented above.

The titratable acidity of the single fermentation SC group was 5.91 g/L, with a pH of 3.88; for the YC-T7+SC group, it was 5.31 g/L and pH 4.02; and for the QC-P3+SC group, it was 5.73 g/L and pH 3.97. The total phenolic content reached 3.48 g/L in the YC-T7+SC group and 3.45 g/L in the QC-P3+SC group, both significantly higher than the 3.09 g/L observed in the single fermentation SC group, likely due to NS metabolic activity promoting the release of total polyphenols [21]. The results indicate that co-fermentation of native non-*Saccharomyces* yeasts from Xinjiang with SC effectively improved the acidity balance and increased the total phenolic content.

#### 3.3.3. Organic Acid Contents

The organic acid results, as shown in Table 3, indicate that mixed-strain fermentation significantly altered the organic acid profile of the wine samples, with tartaric acid, citric acid, and α-ketoglutaric acid all increasing notably compared to single SC fermentation. Tartaric acid, the core structural component of wine acidity, reached concentrations of 1778.27 mg/L and 1496.35 mg/L in the QC-P3+SC and YC-T7+SC groups, respectively—representing increases of 22.58% and 3.15% over the SC group’s 1450.69 mg/L, with the QC-P3+SC group showing the most significant rise. This enhancement strengthens the wine’s acidity structure and improves color and microbial stability. Citric acid levels also increased, reaching 419.06 mg/L and 373.31 mg/L in the QC-P3+SC and YC-T7+SC groups, respectively—14.7% and 2.18% higher than the SC group’s 365.36 mg/L. α-ketoglutarate is an intermediate in the tricarboxylic acid cycle and participates in amino acid metabolism via the glutamate dehydrogenase reaction [22]. It reached 96.02 mg/L in the QC-P3+SC group and 71.02 mg/L in the YC-T7+SC group, representing increases of 51.64% and 12.16% respectively compared with 63.32 mg/L in the SC group. The accumulation of α-ketoglutaric acid may reflect the active amino acid metabolism of NS yeast in the mixed culture fermentation system, and the metabolic conversion of amino acids (e.g., the production of higher alcohols via the Ehrlich pathway) is closely associated with the formation of aroma compounds [23]. Therefore, changes in α-ketoglutarate levels can potentially be used as an indirect indicator for nitrogen metabolic activity in fermentation systems, yet the direct correlation between α-ketoglutarate levels and the biosynthesis of aroma compounds still requires further experimental verification.

Meanwhile, acetic acid levels increased in the mixed-strain groups, reaching 69.28 mg/L and 59.53 mg/L in the YC-T7+SC and QC-P3+SC groups, respectively—76.28% and 51.48% higher than the SC group’s 39.3 mg/L. Nevertheless, these values remain well below national standard limits and do not adversely affect wine hygiene. Lactic acid and pyruvic acid levels showed slight reductions. The QC-P3+SC group exhibited greater increases in tartaric acid, citric acid, and α-ketoglutaric acid compared to the YC-T7+SC group, while the latter showed relatively higher acetic acid accumulation, indicating that NS’s regulation of organic acid metabolism is strain-specific. Overall, mixed-strain fermentation optimizes the physicochemical and sensory qualities of Munage grape wine by synergistically enhancing tartaric acid, citric acid, and α-ketoglutaric acid, thereby improving the acidity structure, taste balance, and aromatic precursors.

### 3.4. Analysis of Volatile Aroma Compounds

#### 3.4.1. Composition of Volatile Aroma Compounds in Munage Wine

As shown in Table 4, a total of 54 volatile compounds belonging to five categories, including esters, alcohols, acids, phenols and other compounds, were detected in all samples. The T7+SC group exhibited relatively higher acetic acid accumulation, indicating that the impact of regulation of NS on organic acid metabolism also presents strain specificity. Overall, through the synergistic increase in the contents of tartaric acid, citric acid and α-ketoglutaric acid, mixed-culture fermentation optimized the physicochemical and sensory properties of Munage grape wine from three aspects: acid structure, taste harmony and aroma precursors.

In the SC, YC-T7+SC, and QC-P3+SC groups, 49, 48, and 50 volatile compounds were detected, respectively. Esters were the most abundant [24], with 27, 26, and 27 detected in the three groups, respectively; followed by alcohols, with 12 detected in the QC-P3+SC group and 11 detected in the other two groups; for acids, 7 were detected in the QC-P3+SC group and 6 were detected in the other two groups; for phenols, 3 were detected in both the SC and YC-T7+SC groups, while 2 were detected in the QC-P3+SC group; and 2 other volatile compounds were detected in all groups.

#### 3.4.2. Volatile Aroma Compounds in Munage Wine

As shown in Table 5, a total of 54 volatile components were detected, including 29 esters, 12 alcohols, 7 acids, 4 phenols and 2 other components.

The results show that among all tested samples, ethyl 9-decenoate has the highest content, followed by isoamyl acetate, ethyl butyrate, isoamyl hexanoate and ethyl decanoate. In terms of acetate analysis, the contents of ethyl acetate in the YC-T7+SC group and the QC-P3+SC group were 791.71 μg/L and 831.00 μg/L, respectively, representing 1.23-fold and 1.29-fold increases compared with that in the SC group. The contents of isoamyl acetate in the YC-T7+SC group and the QC-P3+SC group were 5294.57 μg/L and 4420.76 μg/L, respectively, which were 1.47-fold and 1.22-fold higher than that in the SC group. In addition, the content of hexyl acetate in the YC-T7+SC group was 337.93 μg/L, compared with 286.02 μg/L in the SC group, representing a 1.18-fold increase. With respect to ethyl ester analysis, the contents of ethyl hexanoate in the YC-T7+SC group and the QC-P3+SC group were 1294.92 μg/L and 1048.66 μg/L, respectively, showing 1.68-fold and 1.36-fold increases relative to the SC group. The contents of ethyl octanoate in the YC-T7+SC group and the QC-P3+SC group were 255.86 μg/L and 132.29 μg/L, which increased by 3.54-fold and 1.83-fold respectively compared with the SC group. The content of ethyl nonanoate in the YC-T7+SC group was 22.35 μg/L, while that in the SC group was 16.09 μg/L, representing a 1.39-fold increase. It is worth noting that ethyl butyrate was only detected in the QC-P3+SC group at a content of 4234.45 μg/L, which enriched the ester profile of the product. In conclusion, mixed-culture fermentation effectively increases the content of ester compounds [25].

In this study, 12 alcohol compounds were detected. The isoamyl alcohol content was 6782.03 μg/L in the YC-T7+SC group and 5861.92 μg/L in the QC-P3+SC group. Compared with the SC control group, the isoamyl alcohol content in the YC-T7+SC group increased by 1.12 times, while that in the QC-P3+SC group decreased to 0.97 times that of the SC group. The n-hexanol content was 145.96 μg/L in the YC-T7+SC group and 127.48 μg/L in the QC-P3+SC group; compared with the SC group, the n-hexanol content in the YC-T7+SC group decreased to 0.62 times that of the SC group, and that in the QC-P3+SC group decreased by 0.54 times relative to the SC group. The n-heptanol content was 710.13 μg/L in the YC-T7+SC group and 472.94 μg/L in the QC-P3+SC group; compared with the SC group, the n-heptanol content in the YC-T7+SC group increased by 2.62 times, and that in the QC-P3+SC group increased by 1.74 times. The nerolidol content was 3490.61 μg/L in the YC-T7+SC group and 2978.92 μg/L in the QC-P3+SC group; compared with the SC group, the nerolidol content in the YC-T7+SC group increased by 1.12 times, while that in the QC-P3+SC group decreased to 0.95 times that of the SC group. These results indicate that the YC-T7+SC treatment increases the total content of alcohol compounds, while the QC-P3+SC treatment reduces the content of alcohol compounds [26].

The primary function of acidic substances is to balance the aroma of the flavor [27]. In the SC group, the octanoic acid content was 1431.57 μg/L; in the YC-T7+SC group, the octanoic acid content reached 1830.48 μg/L, representing a 1.28-fold increase compared with the SC group; and in the QC-P3+SC group, the octanoic acid content was 1557.65 μg/L, which was 1.09 times higher than that of the SC group. Mixed-culture fermentation increases the content of organic acids, which not only helps prevent the negative effects caused by microbial spoilage and oxidation but also promotes the synthesis of ester compounds, which is critical for the formation of the aroma characteristics of wine bodies [28].

Ketones, ethers, and heterocyclic compounds can harmonize the overall aroma and enrich the flavor profile of the wine. Among them, damascenone is a key aromatic compound with a characteristic rose-like fragrance, with concentrations of 123.29 μg/L in the SC group, 124.86 μg/L in the YC-T7+SC group, and 120.21 μg/L in the QC-P3+SC group.

### 3.5. OAV Analysis

The odor activity value (OAV) is an evaluation indicator for the odor contribution of individual aromatic compounds [29]. As a core indicator reflecting the actual contribution of odor-active compounds to wine aroma, compounds with OAVs greater than 0.1 can exert a significant influence on the aroma profile of wine. Higher OAVs correspond to a stronger odor contribution. As shown in Table 6, a total of 18 key odor-active compounds with OAVs > 0.1 were detected across all tested samples, covering esters, alcohols, acids and terpenes, among which esters are the core components contributing to wine aroma.

As shown in Table 6, there are 18 key aroma compounds with OAVs greater than 0.1. Ethyl caproate exhibits the highest OAV, which reaches 92.49 in the YC-T7+SC group, representing a 68% increase compared with the SC group; the OAV of ethyl caproate in the QC-P3+SC group is 74.91, showing a 36% increase relative to the SC group. In the YC-T7+SC group, the OAVs of isoamyl acetate and isoamyl caproate are 26.47 and 19.82, corresponding to increases of 46% and 22% respectively compared with the SC group. In the QC-P3+SC group, the OAVs of isoamyl acetate and isoamyl caproate are 22.10 and 16.38, which increase by 45% and 20% respectively compared with the SC group. The OAV of phenylethyl acetate in the YC-T7+SC group is 8.57, which is higher than 6.36 in the SC group and 6.15 in the QC-P3+SC group, making a positive contribution to enhancing the floral note hierarchy. Ethyl butyrate, a unique component exclusive to the QC-P3+SC group, has an extremely high OAV of 211.72, far exceeding its sensory threshold, endowing this group with a unique pineapple fruit aroma characteristic.

#### 3.5.1. PCA of Munage Wine

**Figure 6:**
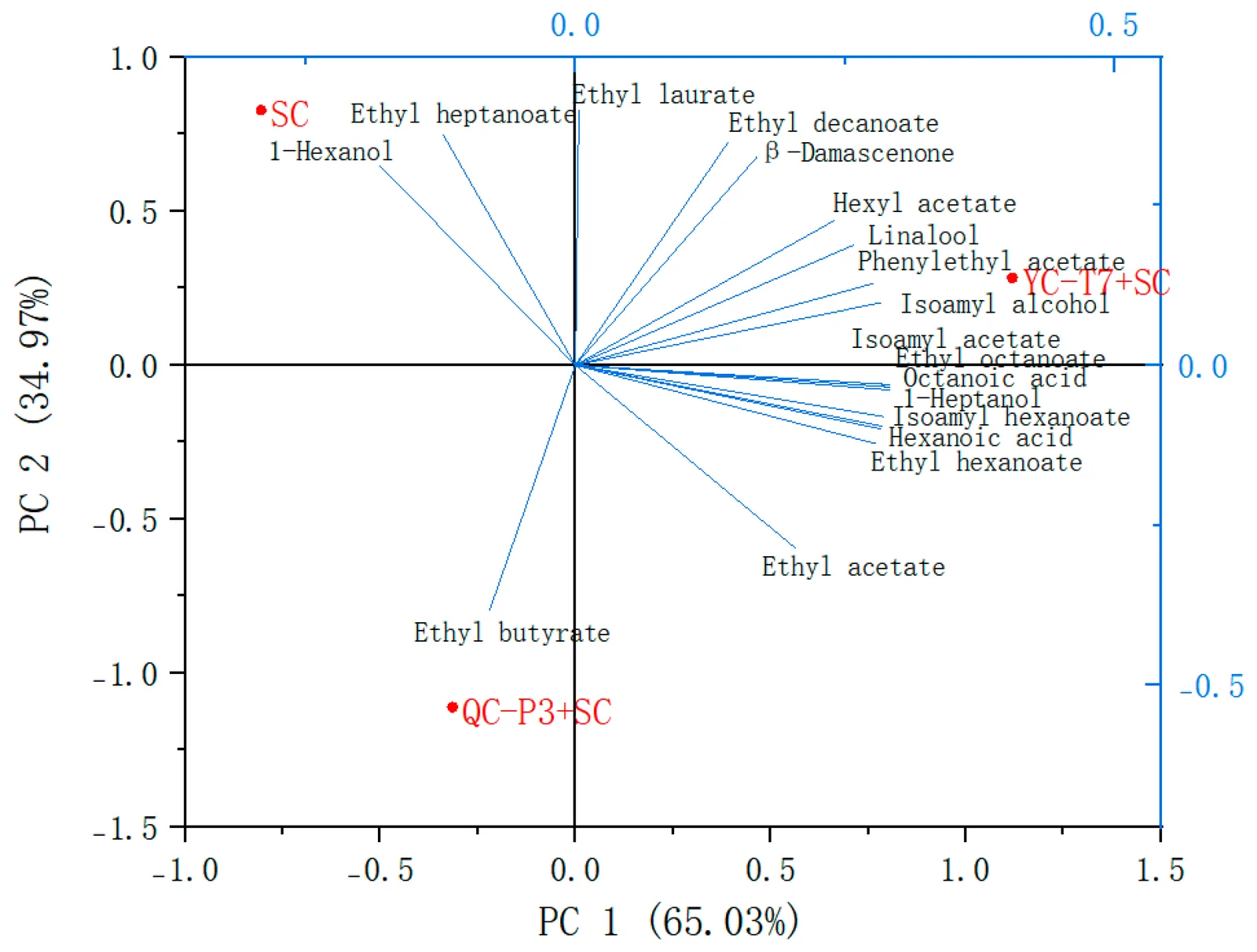
Principal component analysis (PCA) based on 18 key aroma compounds (OAV > 0.1) in Munage wine samples. PC1 and PC2 accounted for 65.03% and 34.97% of total variance, respectively.

To intuitively analyze the effect of mixed-culture fermentation on the aroma profile of wine, 18 aroma compounds with odor activity values (OAVs) greater than 0.1 were selected for principal component analysis (PCA). As shown in Figure 6, the variance contribution rates of PC1 and PC2 were 65.03% and 34.97%, respectively, and the two principal components accounted for all the variation information among samples. YC-T7+SC was located in the first quadrant (positive direction of PC1), showing a strong correlation with ethyl hexanoate, isoamyl hexanoate, octanoic acid, hexanoic acid, linalool, phenethyl acetate, isoamyl alcohol and other compounds; QC-P3+SC was located near the fourth quadrant (negative direction of PC1 and negative direction of PC2), with a strong correlation with ethyl butyrate and ethyl acetate; SC was located in the second quadrant (negative direction of PC1 and positive direction of PC2), and was strongly correlated with ethyl heptanoate, 1-hexanol, ethyl laurate and other compounds. The three groups of samples were distributed in different quadrants in the PCA biplot, with relatively large distances between each other, which further verified the quantitative analysis results presented above: the YC-T7+SC group had the highest content of fruit and floral esters; the QC-P3+SC group was characterized by the unique presence of ethyl butyrate; and the aroma profile of the single-culture fermentation group (SC) was significantly different from that of the mixed-culture fermentation groups [30].

#### 3.5.2. Heatmap Analysis of Munage Wine

**Figure 7:**
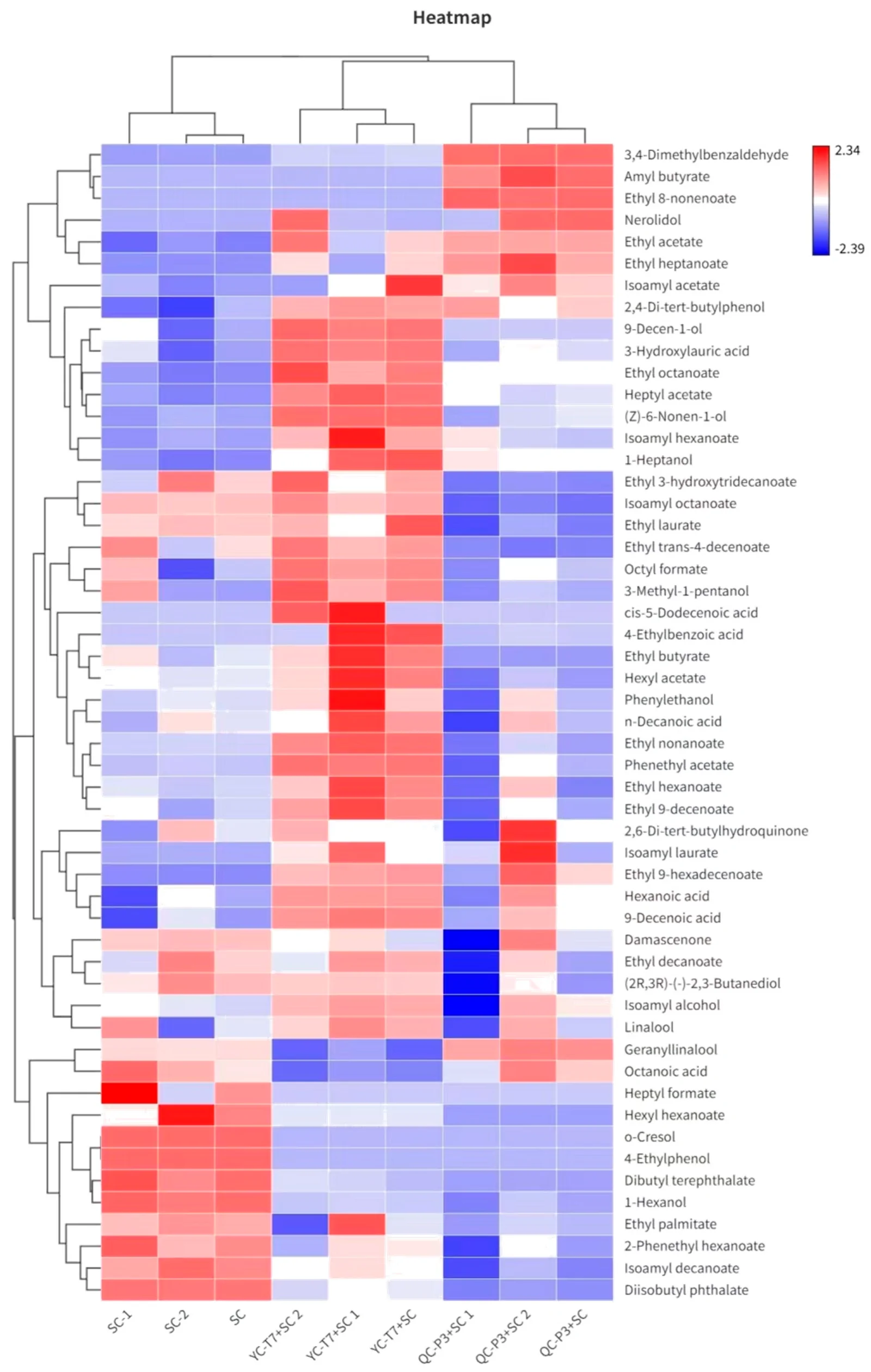
Heatmap of volatile aroma compound contents in Munage wine samples (color intensity indicates relative concentration).

Figure 7 presents the heatmap of volatile aroma compound contents in all samples, where deeper crimson indicates higher compound contents and deeper green represents lower contents. Overall analysis shows that the contents of esters and alcohols in the YC-T7+SC group are relatively high, indicating that YC-T7 can effectively promote the accumulation of fruit and floral aromas; in the QC-P3+SC group, characteristic aroma compounds that impart floral and pineapple notes, such as nerolidol, ethyl heptanoate and amyl butyrate, are more enriched [31].

### 3.6. Sensory Evaluation

**Figure 8:**
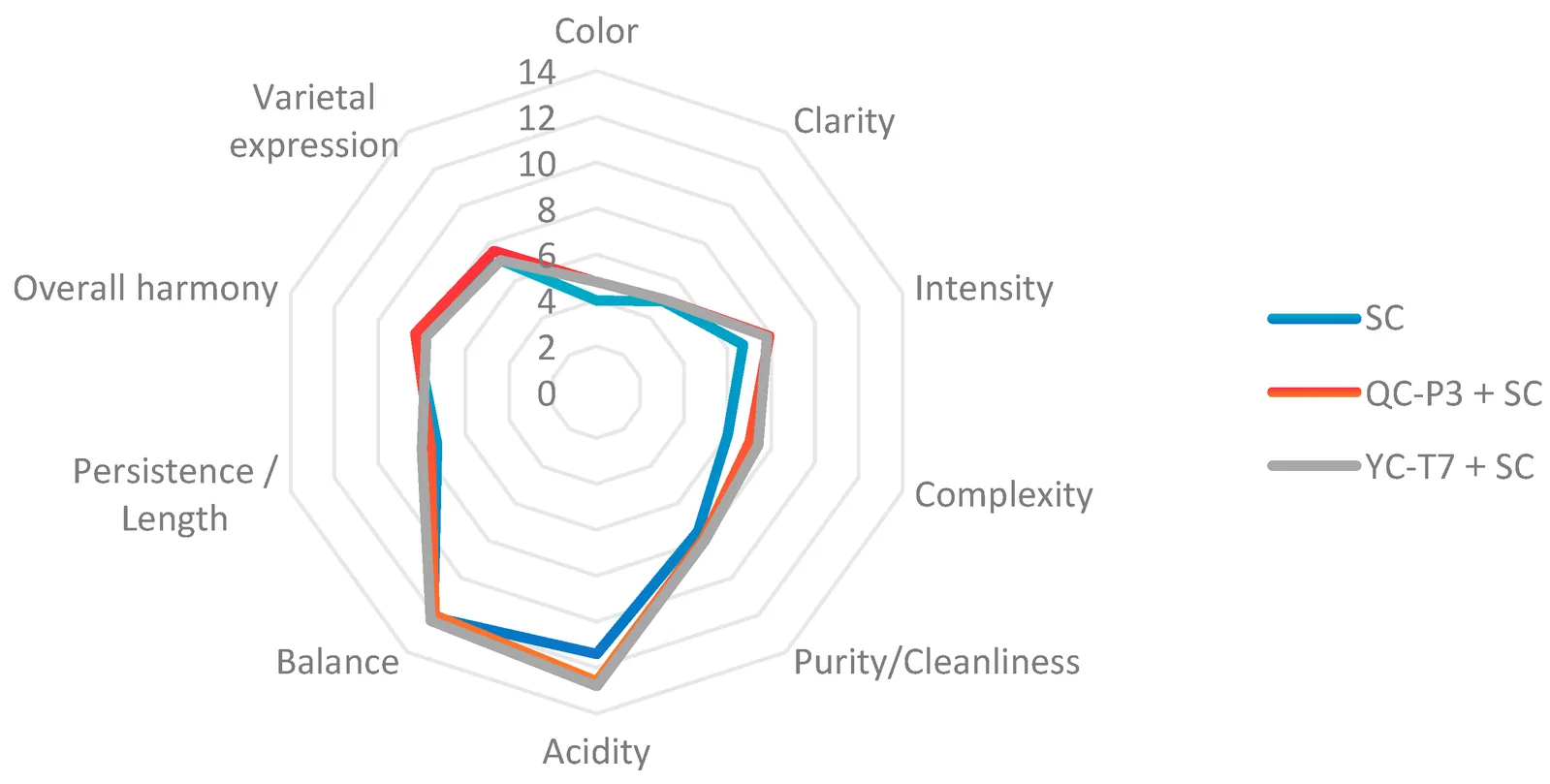
Sensory evaluation radar chart of Munage wine.

Sensory evaluation was performed on three groups of samples. As shown in Figure 8, the YC-T7+SC group obtained the highest score, followed by the QC-P3+SC group, and the SC group had the lowest score. In terms of aroma, the scores of the YC-T7+SC group in aroma intensity and complexity were higher than those of the SC group, which is consistent with the results of the increased content of core esters and aroma contribution of this group in the aforementioned OAV analysis [32]. The QC-P3+SC group had an outstanding score in typical style, which may be consistent with the unique fruity aroma attribute brought by the specific ethyl butyrate in this group [33]. In terms of taste, the mixed-culture fermentation groups outperformed the SC group in balance, aftertaste and overall harmony, which agrees with the physicochemical results presented earlier showing that mixed-culture fermentation reduces titratable acidity and increases the total polyphenol content. The three groups of samples were essentially identical in color, clarity, purity and acidity, indicating that mixed-culture fermentation does not exert adverse effects on the basic sensory quality of wine [34].

## 4. Discussion

This study investigated the mixed fermentation of Mynag grape wine using native non-*Saccharomyces* yeasts YC-T7 and QC-P3 in combination with *S. cerevisiae* CEC01 under sequential inoculation, exploring their co-fermentation characteristics and effects on wine quality.

Previous research has shown that sequential inoculation with the non-*Saccharomyces* yeast *Lachancea thermotolerans* and *S. cerevisiae* can enhance wine acidity and improve balance, mouthfeel, and aroma [35]. Ophélie Dutraive et al. [36] conducted sequential inoculation fermentations of Riesling wine using *Torulaspora delbrueckii*, *Metschnikowia pulcherrima*, *Lachancea thermotolerans*, *Schizo saccharomyces pombe*, and *S. cerevisiae*, comparing them to single *S. cerevisiae* fermentation. They found that sequential inoculation not only modulated wine aroma but also influenced key quality parameters such as glycerol, ethanol, alcohol content, acidity, and fermentation by-products. In this study, sequential inoculation of YC-T7 and QC-P3 with *S. cerevisiae* demonstrated a superior performance over simultaneous inoculation in terms of biomass growth, sugar consumption efficiency, and alcoholic fermentation efficiency.

In this study, mixed fermentation of non-*Saccharomyces* yeast with *S. cerevisiae* revealed that, compared with the single fermentation of *S. cerevisiae*, the total ester content in the YC-T7+SC and QC-P3+SC treatment groups increased by 27.68% and 20.23% respectively, with particularly significant accumulation of ethyl butyrate, ethyl hexanoate, ethyl octanoate and isoamyl acetate. This conferred a prominent pear and pineapple aroma to Munage grape wine. From the perspective of biochemical mechanism, ester synthesis is primarily catalyzed by alcohol acetyltransferases (encoded by the ATF1 and ATF2 genes), which promote the reaction between alcohols and acetyl-CoA to generate esters [27]. It is possible that the mixed fermentation system of non-*Saccharomyces* yeast and *S. cerevisiae* exhibits a stronger capacity for providing ester precursors. In this system, glucose enters the pyruvate metabolic branch, and key enzymes including alcohol dehydrogenase, aminotransferase, acyl-CoA synthetase and esterase sequentially generate important precursor substances, namely higher alcohols, acetyl-CoA and medium-chain acyl-CoA. Through metabolic pathways such as the Ehrlich pathway, fatty acid metabolism and esterification, these precursors collaboratively produce fruit-flavor-imparting secondary metabolites, including isoamyl acetate, ethyl hexanoate and ethyl octanoate. Liu Yang et al. [37] inoculated *Oenococcus oeni* ZX-1 for malolactic fermentation during the alcoholic fermentation of Chardonnay wine, and observed significant accumulation of fatty acid ethyl esters such as ethyl hexanoate, which enhanced the tropical fruit and citrus aroma of the wine; Hongchuan Xia et al. [38] adopted mixed fermentation of *Hanseniaspora uvarum* YUN268 and *Pichia fermentans* Z9Y-3 for Italian Riesling, and found that compared with single fermentation, sequential inoculation and simultaneous inoculation increased the total ester content by 49.4% and 56.5% respectively. The increased ester content in the QC-P3+SC group is in agreement with Wang et al. [39], who demonstrated that *P. kudriavzevii* co-fermentation with *S. cerevisiae* significantly enhanced ethyl butyrate and isoamyl acetate in kiwifruit wine.

Munage grape is rich in conjugated terpene aroma precursors (such as glycosylated linalool and geraniol), which can be further hydrolyzed by β-glucosidase to release free volatile aromas [40]. In this study, the mixed-strain fermentation groups showed that the content of nerolidol in the YC-T7+SC group was 3490.61 μg/L, while in the QC-P3+SC group, it was 2978.92 μg/L. Compared with the SC group, the YC-T7+SC group increased by 1.12-fold, whereas the QC-P3+SC group decreased to 0.95-fold. The content of damascenone was 123.29 μg/L in the SC group, 124.86 μg/L in the YC-T7+SC group, and 120.21 μg/L in the QC-P3+SC group, which may be related to the β-glucosidase activity secreted by non-*Saccharomyces* (NS) yeast. Previous studies have shown that both *P. kudriavzevii* and *K. marxianus* possess extracellular β-glucosidase activity, enabling them to hydrolyze glycosidic bonds and release bound terpenes.

Although differences exist in grape variety, yeast strains, inoculation methods, and fermentation processes, these findings consistently indicate that mixed fermentation with non-*Saccharomyces* yeasts enhances the aromatic compound content and improves the wine quality. This study demonstrates that combining native Xinjiang non-*Saccharomyces* yeasts with *S. cerevisiae* in Munage wine fermentation effectively enriches the types and concentrations of aroma compounds, providing valuable insights for targeted aroma modulation in regionally distinctive grape varieties.

## 5. Conclusions

This study used single *S. cerevisiae* CEC01 fermentation as the control group (SC), and two native non-*Saccharomyces* yeasts, *K. marxianus* YC-T7 and *P. kudriavzevii* QC-P3, from Xinjiang co-inoculated sequentially with SC as the experimental groups (YC-T7+SC, QC-P3+SC), systematically analyzing the effects of mixed-strain fermentation on the quality of Munage wine. The results demonstrated that sequential inoculation significantly enhanced the biomass accumulation and glucose consumption efficiency of non-*Saccharomyces* (NS) strains compared with simultaneous inoculation, while ensuring the smooth completion of alcoholic fermentation. The presence of *Saccharomyces cerevisiae* metabolites did not inhibit the growth and fermentation activity of NS strains, which confirms the feasibility of the sequential inoculation strategy. In sequential fermentation of Munage grape wine, both tested NS strains effectively increased the contents of tartaric acid, citric acid, α-ketoglutaric acid and total polyphenols in the wine, and significantly enriched the variety and content of aroma compounds including esters and terpenes. Specifically, the YC-T7+SC co-fermentation group was characterized by fruit-flavored esters such as ethyl hexanoate and isoamyl acetate, delivering the optimal sensory intensity and hierarchical structure; the QC-P3+SC group presented a unique pineapple fruit aroma attributed to ethyl butyrate, exhibiting a prominent typical style. This study demonstrates that indigenous non-*Saccharomyces* cerevisiae strains from Xinjiang exhibit application potential in the stylized winemaking of Munage grapes, and provides excellent indigenous strain resources and a theoretical basis for the development of region-specific characteristic wines.

## Figures and Tables

**Table 1:** Biomass dynamics (CFU/mL) of single and co-cultures under different inoculation strategies in synthetic grape juice (SGJ) at 28 °C.

Strain/Inoculation Strategy	1 d	2 d	3 d	4 d	5 d	6 d	7 d	8 d	9 d
YC-T7 (single)	1.48 × 10^6^	3.47 × 10^6^	1.26 × 10^7^	4.57 × 10^7^	3.24 × 10^7^	2.04 × 10^7^	1.20 × 10^7^	—	—
QC-P3 (single)	1.58 × 10^6^	3.55 × 10^6^	6.17 × 10^6^	2.63 × 10^7^	2.24 × 10^7^	1.35 × 10^7^	8.71 × 10^6^	—	—
*S. cerevisiae* CEC01 (single)	2.09 × 10^6^	3.63 × 10^6^	8.32 × 10^6^	1.86 × 10^7^	2.69 × 10^7^	2.14 × 10^7^	1.82 × 10^7^	—	—
YC-T7 + SC (simultaneous)	1.86 × 10^6^	4.37 × 10^6^	9.33 × 10^6^	2.82 × 10^7^	1.78 × 10^7^	1.38 × 10^7^	7.94 × 10^6^	—	—
QC-P3 + SC (simultaneous)	2.69 × 10^6^	5.50 × 10^6^	9.12 × 10^6^	1.70 × 10^7^	2.34 × 10^7^	1.66 × 10^7^	1.00 × 10^7^	—	—
YC-T7 + SC (sequential)	1.74 × 10^6^	4.27 × 10^6^	1.51 × 10^7^	3.02 × 10^7^	3.89 × 10^7^	3.47 × 10^7^	2.69 × 10^7^	2.00 × 10^7^	1.17 × 10^7^
QC-P3 + SC (sequential)	1.58 × 10^6^	3.09 × 10^6^	1.05 × 10^7^	1.95 × 10^7^	2.19 × 10^7^	1.86 × 10^7^	1.58 × 10^7^	1.32 × 10^7^	9.77 × 10^6^

**Table 2:** Basic physicochemical indicators of Munage wine fermented at 18 °C by sequential inoculation.

Physicochemical Parameter	SC	YC-T7 + SC	QC-P3 + SC
Ethanol content/%vol	10.62 ± 0.01 a	10.68 ± 0.16 a	10.74 ± 0.18 a
Titratable acidity/g·L^−1^	5.91 ± 0.09 a	5.31 ± 0.13 b	5.73 ± 1.06 a
Volatile acidity/g·L^−1^	0.34 ± 0.01 b	0.53 ± 0.04 a	0.54 ± 0.18 a
pH	3.88 ± 0.01 a	4.02 ± 0.01 a	3.97 ± 0.08 a
Total polyphenols/g·L^−1^	3.09 ± 0.13 b	3.48 ± 0.01 a	3.45 ± 0.01 a
Total sugars/g·L^−1^	1.55 ± 0.07 b	2.25 ± 0.35 a	2.30 ± 0.28 a

Notes: Data are presented as mean ± standard deviation (*n* = 3). Different lowercase letters within the same row indicate significant differences (*p* < 0.05, Duncan’s test). SC, single *S. cerevisiae* CEC01 fermentation; YC-T7+SC, sequential inoculation of *K. marxianus* YC-T7 and *S. cerevisiae* CEC01; QC-P3+SC, sequential inoculation of *P. kudriavzevii* QC-P3 and *S. cerevisiae* CEC01.

**Table 3:** Organic acid contents (mg/L) of Munage wine fermented at 18 °C by sequential inoculation.

Organic Acid	QC-P3 + SC	YC-T7 + SC	SC
Lactic acid	221.46 ± 0.29 a	224.53 ± 29.41 a	229.75 ± 5.57 a
Acetic acid	59.53 ± 24.92 a	69.28 ± 13.91 a	39.30 ± 0.57 a
Pyruvic acid	26.89 ± 1.63 a	26.59 ± 3.03 a	32.32 ± 6.03 a
Tartaric acid	1778.27 ± 29.33 a	1496.35 ± 96.61 b	1450.69 ± 16.62 b
α-Ketoglutaric acid	96.02 ± 7.54 a	71.02 ± 0.64 b	63.32 ± 6.45 b
Citric acid	419.06 ± 24.08 a	373.31 ± 0.09 b	365.36 ± 0.23 b

Notes: Data are presented as mean ± standard deviation (*n* = 3). Different lowercase letters within the same row indicate significant differences (*p* < 0.05, Duncan’s test). SC, single *S. cerevisiae* CEC01 fermentation; YC-T7+SC, sequential inoculation of *K. marxianus* YC-T7 and *S. cerevisiae* CEC01; QC-P3+SC, sequential inoculation of *P. kudriavzevii* QC-P3 and *S. cerevisiae* CEC01.

**Table 4:** Number of volatile substance categories in Munage wine.

Yeast Strain	Esters	Alcohols	Acids	Phenols	Others	Total
SC	27	11	6	3	2	49
YC-T7 + SC	26	11	6	3	2	48
QC-P3 + SC	27	12	7	2	2	50

**Table 5:** Volatile aroma compounds (μg/L) identified in Munage wine samples fermented at 18 °C under different sequential inoculation strategies.

Category	Compound	SC	YC-T7 + SC	QC-P3 + SC	Threshold	Odor Description
Esters	Ethyl acetate	645.77 ± 30.74 a	791.72 ± 112.46 a	831.00 ± 0.00 a	7500	Nail polish, fruity, ester
Esters	Ethyl butyrate	0.00 ± 0.00 b	0.00 ± 0.00 b	4234.45 ± 893.01 a	20	Sour fruit, strawberry, green apple
Esters	Amyl butyrate	137.49 ± 10.21 a	160.05 ± 18.45 a	125.42 ± 0.00 a	—	—
Esters	ethyl 9-decenoate	3613.62 ± 232.74 a	5294.57 ± 1508.94 a	4420.76 ± 199.42 a	200	Banana, fruity, floral, sweet
Esters	Ethyl hexanoate	769.33 ± 69.09 a	1294.92 ± 309.70 a	1048.68 ± 69.76 a	14	Strong fruity and winy
Esters	Hexyl acetate	286.02 ± 3.93 a	337.93 ± 44.95 a	257.87 ± 21.85 a	45	Apple, anise
Esters	Ethyl heptanoate	40.25 ± 28.15 a	13.54 ± 0.49 a	0.00 ± 0.00 a	2.2	Pineapple
Esters	Heptyl acetate	10.38 ± 1.59 a	36.12 ± 17.36 a	32.90 ± 4.57 a	—	—
Esters	Ethyl octanoate	72.33 ± 19.94 b	255.86 ± 23.92 a	132.29 ± 17.89 b	580	Pear, pineapple, floral, fruity
Esters	Isoamyl acetate	2739.21 ± 126.85 b	3963.09 ± 343.44 a	3275.22 ± 0.00 b	200	Fruity, banana
Esters	Ethyl nonanoate	16.09 ± 3.48 a	22.35 ± 3.46 a	12.24 ± 5.05 a	1300	Fruity, rose
Esters	Ethyl 8-nonenoate	58.45 ± 0.40 b	84.18 ± 4.81 a	52.15 ± 9.67 b	—	—
Esters	Octyl formate	0.00 ± 0.00 b	0.00 ± 0.00 b	11.83 ± 0.37 a	—	Fruity
Esters	Heptyl formate	25.18 ± 5.28 a	30.50 ± 1.06 a	25.15 ± 2.63 a	—	—
Esters	Hexyl hexanoate	223.25 ± 299.96 a	0.00 ± 0.00 a	0.00 ± 0.00 a	—	Herbal, vegetative
Esters	Ethyl decanoate	2477.26 ± 566.40 a	2694.52 ± 24.88 a	1808.02 ± 950.13 a	1047	Fatty, fruity, waxy, vinegar
Esters	Isoamyl octanoate	141.94 ± 6.87 a	150.72 ± 14.26 a	94.26 ± 1.29 b	—	Fruity
Esters	Ethyl decanoate (isomer)	4547.98 ± 518.06 a	5683.58 ± 616.15 a	4232.03 ± 719.99 a	—	Fruity
Esters	Ethyl trans-4-decenoate	4.50 ± 1.34 a	5.26 ± 0.57 a	2.74 ± 0.15 a	—	—
Esters	Phenylethyl acetate	1588.75 ± 23.19 a	2142.13 ± 15.89 a	1536.49 ± 350.32 a	250	Floral, peach
Esters	Ethyl laurate	1381.64 ± 74.93 a	1184.71 ± 52.88 a	657.96 ± 257.10 b	1500	Floral-fruity, creamy
Esters	Isoamyl laurate	31.87 ± 38.49 a	13.10 ± 0.26 a	10.76 ± 3.95 a	—	—
Esters	Isoamyl decanoate	149.21 ± 16.91 a	109.25 ± 11.26 a	56.84 ± 30.44 b	—	Fruity
Esters	2-Phenylethyl hexanoate	9.41 ± 1.10 a	7.08 ± 1.35 a	5.91 ± 2.02 a	—	Sweet honey
Esters	Ethyl 3-hydroxytridecanoate	21.29 ± 1.93 a	21.93 ± 1.55 a	18.77 ± 0.34 a	—	—
Esters	Ethyl palmitate	78.03 ± 2.72 a	70.68 ± 21.63 a	64.24 ± 3.85 a	1500	Waxy, fruity, creamy
Esters	Ethyl 9-hexadecenoate	2.38 ± 0.35 c	6.74 ± 0.67 b	8.29 ± 0.00 a	—	Peach, apple or pear
Esters	Phthalic acid diisobutyl ester	9.81 ± 0.06 a	6.63 ± 0.62 b	4.73 ± 0.44 c	—	Aromatic
Esters	Dibutyl phthalate	19.78 ± 3.27 a	6.53 ± 1.00 b	4.64 ± 0.27 b	—	—
**Subtotal**		19,101.22 ± 2087.99	24,387.70 ± 3152.01	22,965.66 ± 3544.51	—	—
Higher alcohols	Isoamyl alcohol	6050.24 ± 692.05 a	6782.03 ± 297.17 a	5861.92 ± 1216.06 a	30,000	Aromatic profile
Higher alcohols	3-Methyl-1-pentanol	22.74 ± 2.61 a	27.74 ± 7.80 a	24.29 ± 5.56 a	—	—
Higher alcohols	1-Hexanol	236.23 ± 7.89 a	145.96 ± 3.90 b	127.48 ± 24.95 b	1100	Winy, ester, fatty
Higher alcohols	2-Octanol	240.00 ± 0.00 a	240.00 ± 0.00 a	240.00 ± 0.00 a	—	—
Higher alcohols	1-Heptanol	271.16 ± 41.69 c	710.13 ± 25.17 a	472.94 ± 16.18 b	2500	Citrus
Higher alcohols	Linalool	10.18 ± 1.59 a	11.06 ± 0.39 a	10.12 ± 1.37 a	25	Fruity
Higher alcohols	Geranyllinalool	17.99 ± 0.38 b	0.00 ± 0.00 c	25.31 ± 2.12 a	—	—
Higher alcohols	(Z)-6-Nonen-1-ol	0.00 ± 0.00 b	18.62 ± 3.47 a	18.62 ± 3.47 a	—	—
Higher alcohols	9-Decen-1-ol	58.74 ± 6.97 c	131.91 ± 0.16 a	80.44 ± 7.65 b	—	—
Higher alcohols	Phenylethyl alcohol	27.79 ± 3.07 a	22.07 ± 6.06 a	17.92 ± 2.63 a	14,000	Rose, honey
Higher alcohols	Nerolidol	3124.03 ± 106.62 a	3490.61 ± 6.49 a	2978.92 ± 634.22 a	—	Spice
Higher alcohols	(2R,3R)-(-)-2,3-Butanediol	34.52 ± 3.02 a	33.53 ± 0.47 a	26.85 ± 9.01 a	120,000	Rubbery chemical, fruity
**Subtotal**		10,093.61 ± 865.88	11,613.65 ± 351.07	9884.83 ± 1923.23	—	—
Acids	Hexanoic acid	107.48 ± 28.04 a	145.98 ± 0.08 a	122.73 ± 33.78 a	420	Cheese, leafy, woody
Acids	Octanoic acid	1431.57 ± 330.77 a	1830.48 ± 157.26 a	1557.65 ± 465.67 a	500	Cheese, astringent
Acids	Decanoic acid	331.60 ± 52.57 a	417.11 ± 86.79 a	305.41 ± 122.86 a	8000	Milky or creamy
Acids	9-Decenoic acid	216.26 ± 0.55 b	402.52 ± 13.24 a	322.01 ± 65.64 a	—	Fruity and milky
Acids	3-Hydroxylauric acid	4.15 ± 0.86 b	6.19 ± 0.12 a	4.67 ± 0.63 a	—	—
Acids	4-Ethylbenzoic acid	25.05 ± 0.30 a	27.04 ± 1.31 a	25.41 ± 5.35 a	—	—
Acids	cis-5-Dodecenoic acid	0.00 ± 0.00 b	0.00 ± 0.00 a	9.21 ± 0.60 b	—	—
**Subtotal**		2116.11 ± 413.09	2829.33 ± 258.79	2347.09 ± 694.53	—	—
Phenols	4-Ethylphenol	0.00 ± 0.00 b	3.87 ± 0.63 a	0.00 ± 0.00 b	—	—
Phenols	2,4-Di-tert-butylphenol	236.51 ± 50.09 a	306.99 ± 9.26 a	288.87 ± 30.04 a	—	Woody, sweet
Phenols	2,6-Di-tert-butylhydroquinone	7.26 ± 1.49 a	7.94 ± 0.77 a	7.66 ± 3.25 a	—	—
**Subtotal**		286.49 ± 51.85	318.80 ± 10.67	296.54 ± 33.29	—	—
Others	3,4-Dimethylbenzaldehyde	40.92 ± 1.88 a	9.93 ± 2.93 b	38.27 ± 3.01 a	—	—
Others	β-Damascenone	123.29 ± 4.87 a	124.86 ± 16.79 a	120.21 ± 30.29 a	50	—
**Subtotal**		164.21 ± 6.75	134.79 ± 19.72	158.48 ± 33.30	—	—

Notes: Data are presented as mean ± standard deviation (*n* = 3). Different lowercase letters within the same row indicate significant differences (*p* < 0.05, Duncan’s test). SC, single *S. cerevisiae* CEC01 fermentation; YC-T7+SC, sequential inoculation of *K. marxianus* YC-T7 and *S. cerevisiae* CEC01; QC-P3+SC, sequential inoculation of *P. kudriavzevii* QC-P3 and *S. cerevisiae* CEC01.

**Table 6:** Odor activity values (OAVs) of key aroma compounds (OAV > 0.1) in Munage wine.

Compound	SC	YC-T7 + SC	QC-P3 + SC	Threshold (μg/L) [15,16,17]
Ethyl acetate	/	0.11	0.11	7500
Ethyl butyrate	/	/	211.72	20
Isoamyl acetate	18.07	26.47	22.10	200
Ethyl hexanoate	54.95	92.49	74.91	14
Hexyl acetate	6.36	7.51	5.73	45
Ethyl heptanoate	18.30	6.15	/	2.2
Ethyl octanoate	0.12	0.44	0.23	580
Isoamyl hexanoate	13.70	19.82	16.38	200
Ethyl decanoate	2.37	2.57	1.73	1047
Phenylethyl acetate	6.36	8.57	6.15	250
Ethyl laurate	0.92	0.79	0.44	1500
Isoamyl alcohol	0.20	0.23	0.20	30,000
1-Hexanol	0.21	0.13	0.12	1100
1-Heptanol	0.11	0.28	0.19	2500
Linalool	0.41	0.44	0.40	25
Hexanoic acid	0.26	0.35	0.29	420
Octanoic acid	2.86	3.66	3.12	500
β-Damascenone	2.47	2.50	2.40	50

Note:”/” indicates that the odor activity value (OAV) was below 0.1 for that compound in the wine.

## Data Availability

The original contributions presented in this study are included in the article. Further inquiries can be directed to the corresponding author.

## Abbreviations

The following abbreviations are used in this manuscript:

NS	Non-*Saccharomyces* yeasts
SC	*Saccharomyces cerevisiae*
SGJ	Synthetic grape juice
YPD	Yeast extract peptone dextrose
HS-SPME-GC-MS	Headspace solid-phase microextraction coupled with gas chromatography–mass spectrometry
OAV	Odor activity value
PCA	Principal component analysis
DVB/CAR/PDMS	Divinylbenzene/carboxen/polydimethylsiloxane
RI	Retention index
CFU	Colony-forming unit

## References

[1] Zhang D., Zhang Y., Lin K., Wang B., Shi X., Cheng W. (2021). Comparison of sugars, organic acids and aroma components of five table grapes in Xinjiang. Earth Environ. Sci..

[2] Zhu W., Zhang W., Qin T., Liao J., Zhang X. (2022). Effects of Purified β-Glucosidases from *Issatchenkia terricola*, *Pichia kudriavzevii*, *Metschnikowia pulcherrima* on the Flavor Complexity and Typicality of Wines. J. Fungi.

[3] Wang J.-Q., Hu J.-L., Feng Q.-Q., Zhang Y., Zhang H.-Y., Zhang S.-F., Li Y.-K., Yang H.-F., Jin G.-J. (2026). Aroma in ‘Munage’ Grape Spirits Response to Different Inoculation Strategies of *H. uvarum/P. fermentans* in Mixed Fermentation with *S. cerevisiae*. Food Biosci..

[4] Ciani M., Comitini F. (2015). Yeast interactions in multi-starter wine fermentation. Curr. Opin. Food Sci..

[5] López-Enríquez L., Genovés S., Furio A.M., Martí N., Ramón D., Serratosa M.P. (2023). Non-*Saccharomyces* Yeasts from Organic Vineyards as Spontaneous Fermentation Agents. Foods.

[6] van Wyk N., Grossmann M., Wendland J., von Wallbrunn C., Pretorius I.S. (2019). The Whiff of Wine Yeast Innovation: Strategies for Enhancing Aroma Production by Yeast during Wine Fermentation. J. Agric. Food Chem..

[7] Li L., Yuan C., Zhang L., Chu R., Yu Q., Cai J., Yang T., Zhang M. (2024). The impact of simultaneous inoculation with *Torulaspora delbrueckii* and *Hanseniaspora uvarum* combined with *Saccharomyces cerevisiae* on chemical and sensory quality of Sauvignon blanc wines. Front. Microbiol..

[8] Chen Y., Lei X., Sun L., Gao B., An P., Ye D., Mu H., Qin Y., Song Y., Liu Y. (2025). Exploring the potentials of indigenous *Saccharomyces cerevisiae* and *Pichia kudriavzevii* for enhancing flavour and aromatic characteristics in apricot wines. Food Chem. X.

[9] Barone E., Ponticello G., Giaramida P., Planeta D., Todaro A., Cassano A., Santangelo F., Leotta G., Figuli E., Moio L. (2021). Use of *Kluyveromyces marxianus* to Increase Free Monoterpenes and Aliphatic Esters in White Wines. Fermentation.

[10] Liu S., Lou Y., Zhao X., Yang X., Li M., Zhang J., Wang Y., Wang X., Fan Y., Huang D. (2024). Multi-omics analyses of the mechanism for formation of key aroma-active compounds in blood orange wine fermented by *Pichia kudriavzevii*. Food Res. Int..

[11] Chen Y., Qi J., Yang H., Lei X., Jiang J., Song Y., Liu Y.L. (2023). Fungal dynamic during apricot wine spontaneous fermentation and aromatic characteristics of *Pichia kudriavzevii* for potential as starter. Food Chem. X.

[12] Ciani M., Capece A., Comitini F., Canonico L., Siesto G., Romano P. (2016). Yeast Interactions in Inoculated Wine Fermentation. Front. Microbiol..

[13] Padilla B., Zulian L., Ferreres Á., Pastor R., Esteve-Zarzoso B., Beltran G., Mas A. (2017). Sequential Inoculation of Native Non-*Saccharomyces* and *Saccharomyces cerevisiae* Strains for Wine Making. Front. Microbiol..

[14] (2006). Analytical Methods of Wine and Fruit Wine.

[15] Noguerol-Pato R., González-Barreiro C., Cancho-Grande B., Simal-Gándara J. (2009). Quantitative Determination and Characterisation of the Main Odourants of Mencía Monovarietal Red Wines. Food Chem..

[16] Fan S., Tang K., Xu Y., Chen S. (2020). Characterization of the potent odorants in Tibetan Qingke Jiu by sensory analysis, aroma extract dilution analysis, quantitative analysis and odor activity values. Food Res. Int..

[17] Ferreira V., López R., Cacho J.F. (2000). Quantitative determination of the odorants of young red wines from different grape varieties. J. Sci. Food Agric..

[18] (2016). Sensory Analysis—Methodology—General Guidance for Establishing a Sensory Profile.

[19] (2007). Sensory Analysis—General Guidance for the Design of Test Rooms.

[20] Rollero S., Bloem A., Ortiz-Julien A., Camarasa C., Divol B. (2018). Altered Fermentation Performances, Growth, and Metabolic Footprints Reveal Competition for Nutrients between Yeast Species Inoculated in Synthetic Grape Juice-Like Medium. Front. Microbiol..

[21] Escribano-Viana R., González-Arenzana L., Portu J., Garijo P., López-Alfaro I., López R., Santamaría P., Gutiérrez A.R. (2018). Wine aroma evolution throughout alcoholic fermentation sequentially inoculated with non-*Saccharomyces*/*Saccharomyces* yeasts. Food Res. Int..

[22] Hinca S.B., Salcedo C., Wagner A., Goldeman C., Sadat E., Aibar M.M.D., Maechler P., Brodin B., Aldana B.I., Helms H.C.C. (2021). Brain endothelial cells metabolize glutamate via glutamate dehydrogenase to replenish TCA-intermediates and produce ATP under hypoglycemic conditions. J. Neurochem..

[23] Curiel J.A., Morales P., Gonzalez R., Tronchoni J. (2017). Different Non-*Saccharomyces* Yeast Species Stimulate Nutrient Consumption in *S. cerevisiae* Mixed Cultures. Front. Microbiol..

[24] Tufariello M., Fragasso M., Pico J., Panighel A., Castellarin S.D., Flamini R., Grieco F. (2021). Influence of Non-*Saccharomyces* on Wine Chemistry: A Focus on Aroma-Related Compounds. Molecules.

[25] Karabegović I., Malićanin M., Stamenković Stojanović S., Lazić M., Smailagić A., Danilović B., Petrović J., Đorđević N., Cvetković D. (2022). Native Yeasts as a Tool to Produce Distinctive and Diverse Tamjanika Grape Wines. Foods.

[26] Lee S.B., Banda C., Park H.D. (2019). Effect of inoculation strategy of non-*Saccharomyces* yeasts on fermentation characteristics and volatile higher alcohols and esters in Campbell Early wines. Aust. J. Grape Wine Res..

[27] Chen L., Darriet P. (2021). Strategies for the Identification and Sensory Evaluation of Volatile Constituents in Wine. Compr. Rev. Food Sci. Food Saf..

[28] Saerens S.M.G., Delvaux F.R., Verstrepen K.J., Thevelein J.M. (2010). Production and biological function of volatile esters in *Saccharomyces cerevisiae*. Microb. Biotechnol..

[29] Francis I.L., Newton J.L. (2005). Determining wine aroma from compositional data. Aust. J. Grape Wine Res..

[30] Muñoz-Castells R., Moreno J., García-Martínez T., Mauricio J.C., Moreno-García J. (2023). Chemometric differentiation of white wines from a low-aromatic grape obtained by spontaneous fermentation, enriched with non-*Saccharomyces*, or with a high-glutathione-producing *Saccharomyces* yeast. Fermentation.

[31] Fu Y., Liu X., Wang J., Li M., Wang Y., Hou J., Zhang X., Li S. (2024). Effects of non-*Saccharomyces* yeasts and their pairwise combinations in co-fermentation with *Saccharomyces cerevisiae* on the quality of Chunjian citrus wine. Molecules.

[32] Ruci M., Kongoli R., Coppola F., Succi M., Testa B., Kyçyk O., Karaulli J., Lamçe F., Iorizzo M. (2025). Preliminary insights regarding the quality of Kallmet wine, obtained by sequential inoculation with *Metschnikowia pulcherrima* and *Saccharomyces cerevisiae*. Front. Microbiol..

[33] Nzoyisaba D., He J., Bao Y., Li R., Li C., Feng B., Lv Y., Liu S. (2025). Improvement of flavor and quality of chardonnay wine by using co-fermentation of non-*Saccharomyces* yeasts and *Saccharomyces cerevisiae*. World J. Microbiol. Biotechnol..

[34] Aplin J.J., White K.P., Edinger A.C., Chacon-Rodriguez L., Runnebaum R.C. (2021). Chemical and Sensory Profiles of Merlot Wines Produced by Sequential Inoculation of *Metschnikowia pulcherrima* or *Meyerzyma guilliermondii*. Fermentation.

[35] Su Y., Seguin P., Belair G., Zhang Z., Li M., Chen S. (2024). Impact of sequential inoculation timing on the quality of wine fermented by indigenous *Lachancea thermotolerans* and *Saccharomyces cerevisiae*. LWT.

[36] Dutraive O., Benito S., Fritsch S., Beisert B., Patz C.-D., Rauhut D. (2019). Effect of Sequential Inoculation with Non-*Saccharomyces* and *Saccharomyces* Yeasts on Riesling Wine Chemical Composition. Fermentation.

[37] Yang L., Zhu X., Mao Y., Zhang J., Zhan C., Liu Y., Li C., Shi Y. (2024). Effect of different inoculation strategies of mixed culture *Saccharomyces cerevisiae*/*Oenococcus oeni* on the aroma quality of Chardonnay wine. Food Res. Int..

[38] Xia H., Zhang Z., Sun L., Wang J., Ma C., Pan Y., Zhao G. (2023). Effects of mixed fermentation on the aroma compounds of ‘Italian Riesling’ dry white wine in eastern foothill of Helan mountain. Fermentation.

[39] Wang Y.-W., Huang Y.-F., Guo Y.-Q., Sun L., Jiang Z.-L., Zhu Y.-T., Zeng R.-Q., Li Q., Xiao C., Zuo Y. (2024). Dissecting Interactions of *Saccharomyces cerevisiae* and *Pichia kudriavzevii* to Shape Kiwifruit Wine Flavor. Foods.

[40] Yuan L., Hou X., He Y., Wan X., Han J., Xu W., Yu S., Bi S., Ma L., Xing L. (2026). Efficient hydrolysis of glycosides by *Pichia kudriavzevii* with high-activity β-glucosidase: A critical role in enhancing terpene aroma in beer. Food Chem..

